# Hippocampal-Prefrontal θ Coupling Develops as Mice Become Proficient in Associative Odorant Discrimination Learning

**DOI:** 10.1523/ENEURO.0259-22.2022

**Published:** 2022-10-05

**Authors:** Daniel Ramirez-Gordillo, K. Ulrich Bayer, Diego Restrepo

**Affiliations:** 1 Department of Neurosurgery, University of Colorado Anschutz Medical Campus School of Medicine, Aurora, CO 80045; 2 Neuroscience Program, University of Colorado Anschutz Medical Campus School of Medicine, Aurora, CO 80045; 3 Department of Pharmacology, University of Colorado Anschutz Medical Campus School of Medicine, Aurora, CO 80045

**Keywords:** learning, memory, neuronal oscillations, odor, phase amplitude coupling, θ reference power

## Abstract

Learning and memory requires coordinated activity between different regions of the brain. Here, we studied the interaction between infralimbic medial prefrontal cortex (mPFC) and hippocampal dorsal CA1 during associative odorant discrimination learning in the mouse. We found that as the animal learns to discriminate odorants in a go-no go task, the coupling of high-frequency neural oscillations to the phase of θ oscillations (θ-referenced phase-amplitude coupling or tPAC) changes in a manner that results in divergence between rewarded and unrewarded odorant-elicited changes in the θ phase-referenced power (tPRP) for β and γ oscillations. In addition, in the proficient animal there was a decrease in the coordinated oscillatory activity between CA1 and mPFC in the presence of the unrewarded odorant. Furthermore, the changes in tPAC resulted in a marked increase in the accuracy for decoding contextual odorant identity from tPRP when the animal became proficient. Finally, we studied the role of Ca^2+^/calmodulin-dependent protein kinase IIα (CaMKIIα), a protein involved in learning and memory, in oscillatory neural processing in this task. We find that the accuracy for decoding the contextual odorant identity from tPRP decreases in CaMKIIα knock-out mice and that this accuracy correlates with behavioral performance. These results implicate a role for tPAC and CaMKIIα in olfactory go-no go associative learning in the hippocampal-prefrontal circuit.

## Significance Statement

Coupling of neural oscillations within and between hippocampal CA1 and medial prefrontal cortex (mPFC) is involved in spatial learning and memory, but the role of oscillation coupling for other learning tasks is not well understood. Here, we performed local field potential (LFP) recording in CA1 and mPFC in mice learning to differentiate rewarded from unrewarded odorants in an associative learning task. We find that odorant-elicited changes in the power of bursts of γ oscillations at distinct phases of θ oscillations become divergent as the animal becomes proficient allowing decoding of contextual odorant identity. Finally, we find that the accuracy to decode contextual odorant identity decreases in mice deficient for the expression of Ca^2+^/calmodulin-dependent protein kinase IIα (CaMKIIα), a protein involved in synaptic plasticity.

## Introduction

Our lives are enhanced, and our personalities are shaped because of the ability to learn and form memories (Klein et al., 2002). Therefore, it is not surprising that often diseases that affect these abilities are devastating and frequently the individual affected becomes dependent on others. Learning and memory requires coordinated activity between different brain regions (Colgin, 2011; Gordon, 2011; Headley and Paré, 2017; Lisman et al., 2017). Local field potential (LFP) oscillations reflect activity of temporally coordinated neuronal groups providing a reference for spike timing-based codes and gating information transfer between distant brain regions. In θ-referenced phase amplitude coupling (tPAC), the amplitude of the bursts of a faster oscillation is larger within a phase window of a slower carrier wave (Tort et al., 2010). Losacco et al. (2020) characterized tPAC in the olfactory bulb (OB) of mice learning to discriminate odorants in a go-no go associative learning task and they introduced a measure of the magnitude of θ phase-locked high γ (65–95 Hz) and β (15–30 Hz) bursts as a function of time: the θ phase-referenced power (tPRP). They showed that tPRP increased for rewarded and decreased for unrewarded odorants in the proficient mouse (Losacco et al., 2020). Furthermore, they showed that contextual odorant identity (is the odorant rewarded?) can be decoded from peak high γ and peak and trough β tPRP in animals proficient in odorant discrimination. These findings in the OB, the first relay station in the olfactory system, raised the question whether downstream areas of the brain experience a similar phenomenon.

Here, we assessed decoding of contextual odorant identity from oscillatory neural activity in animals learning to discriminate odorants in the go-no go task in two downstream brain areas of the brain that receive input from the OB: CA1 of the hippocampus and medial prefrontal cortex (mPFC) involved in learning of odorant valence and attention to odorants (Martin et al., 2007; Gourévitch et al., 2010; Y Li et al., 2017; Wang et al., 2020; Cansler et al., 2022). We chose to survey tPAC and tPRP in CA1 and mPFC because optogenetic modulation of interneurons indicates that θ phase-referenced neural activity is involved in memory encoding and retrieval in CA1 (Siegle and Wilson, 2014). We focused on β and high γ tPRP because strong directional β coupling from the OB to the dorsal hippocampus has been shown to be involved in odor processing in the go-no go task (Gourévitch et al., 2010) and because OB spike-high γ field coherence carries odorant information (A Li et al., 2015), and high γ conveys input from the entorhinal cortex to CA1 (Colgin and Moser, 2010).

Finally, we chose to study odorant decoding in Ca^2+^/calmodulin-dependent protein kinase IIα knock-out mice (CaMKIIα KO) because of the essential role of this protein in learning and memory (Lisman et al., 2012; Bear et al., 2018; Bayer and Schulman, 2019). CaMKIIα plays a role in long-term potentiation (LTP), long-term depression (LTD), and dentate gyrus neurogenesis (Malinow et al., 1989; Silva et al., 1992b; Coultrap et al., 2014; Suárez-Pereira et al., 2015; Cook et al., 2021). Deficiencies in the function of CaMKIIα have been implicated in a range of diseases including schizophrenia, addiction, depression, epilepsy, and neurodevelopmental disorders (Robison, 2014; Chia et al., 2018). CaMKIIα KO are viable and display impaired spatial memory and reduced hippocampal LTP (Silva et al., 1992a, b). Mice heterozygous for CaMKIIα (CaMKIIα Hets) have problems with working memory, increased anxiety, and aggressiveness, characteristic of schizophrenia (Yamasaki et al., 2008; Hasegawa et al., 2009; Matsuo et al., 2009; Chen et al., 1994). Large genome screens have found heterozygous mutations in CaMKIIα in schizophrenia patients (Fromer et al., 2014; Purcell et al., 2014). Furthermore, CaMKIIα Het mice have an immature dentate gyrus (Yamasaki et al., 2008).

## Materials and Methods

See Table 1 for key resources.

**Table 1:** Key resources

Reagent type	Reagent or resource	Source	Identifier	Additional information
Chemical compound, drug	Isoamyl acetate	Sigma-Aldrich	Catalog #123-92-2	Odorant
Chemical compound, drug	Ethyl acetate	Sigma-Aldrich	Catalog #141-78-6	Odorant
Chemical compound, drug	Propyl acetate	Sigma-Aldrich	Catalog #109-60-4	Odorant
Chemical compound, drug	Mineral oil	Sigma-Aldrich	Catalog #8042-47-5	Odorant
Chemical compound, drug	Acetophenone	Sigma-Aldrich	Catalog #98-86-2	Odorant
Chemical compound, drug	Ethyl benzoate	Sigma-Aldrich	Catalog #93-89-0	Odorant
Other	Nickel-chrome wire	Sandvik	Catalog #PX000002	Tetrode fabrication
Other	16 Channel Electrode Interface Board	Neuralynx	EIB-16	Tetrode fabrication
Software, algorithm	Analysis code	This paper	Available on GitHub and by request	
Strain, strain background	Mouse: CaMKIIα WT	Bayer Lab	Coultrap et al. (2014)	Male mice
Strain, strain background	Mouse: CaMKIIα HET	Bayer Lab	Coultrap et al. (2014)	Male mice
Strain, strain background	Mouse: CaMKIIα KO	Bayer Lab	Coultrap et al. (2014)	Male mice
Software, algorithm	MATLAB_R2018a	MathWorks	RRID: SCR_001622	
Software, algorithm	Illustrator	Adobe	RRID: SCR_010279	
Software, algorithm	Photoshop	Adobe	RRID: SCR_014199	

### Animals

Littermate mice with genotypes of CaMKIIα KO, Het, and wild-type (WT) were obtained from heterozygous breeding (Coultrap et al., 2014). Mice were housed in a vivarium with a reversed light cycle of 14/10 h light/dark periods with lights on at 10 P.M. Food (Teklad Global Rodent Diet no. 2918; Harlan) was available *ad libitum*. Access to water was restricted during the behavioral sessions. However, if mice did not obtain ∼1 ml of water during the behavioral session, additional water was provided in a dish in the cage (Slotnick and Restrepo, 2005). All mice were weighed daily and received sufficient water to maintain >80% of the weight before water restriction. All experiments were performed according to protocols approved by the University of Colorado Anschutz Medical Campus Institutional Animal Care and Use Committee.

### Surgery and double tetrode implantation

**Figure 1.**
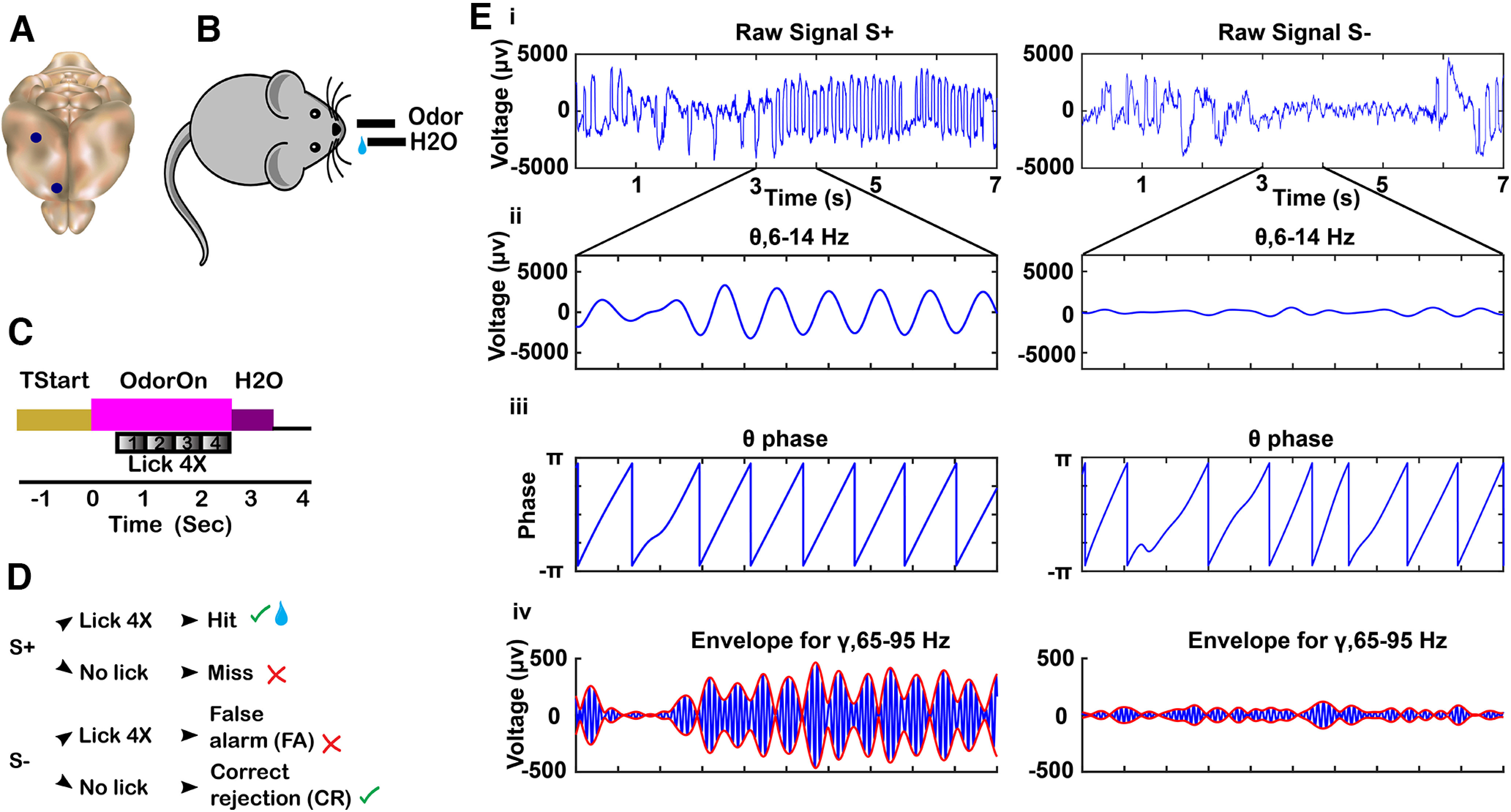
Tetrode location, behavioral task, and tPAC analysis. ***A***, Mouse brain showing location of tetrodes in CA1 and mPFC. ***B***, Mouse undergoing odor discrimination behavioral task. Mouse self-initiates trials by licking the lixit closing the lick detection circuit. Odorants and water were delivered according to the timeline in ***C*** and the decision tree in ***D***. ***C***, Timeline for a single trial. When the animal entered the port and licked at the lixit, it started the trial. TStart (1–1.5 s) is the time from initiation of the trial to odor delivery. At time 0, the final valve air flow was turned toward the odor port for 2.5 s resulting in odor onset ∼100 ms after the valve was activated (Losacco et al., 2020). To obtain the water reward for the rewarded odorant (S+), the mouse must lick at least once during each 0.5-s block for four blocks in the response area. If the stimulus is the rewarded odorant and the animal licked, a water reward was delivered (Hit). If the animal did not lick in each 0.5-s block (Miss), water was not delivered. When the odorant was the unrewarded odorant (S–) the mouse was not rewarded with water regardless of whether it licked in all 0.5-s blocks (false alarm, FA) or not (correct rejection, CR). ***D***, Decision tree for each trial. Green check mark indicates the correct decision was made. Red X mark indicates an incorrect decision was made. Water reward is represented by the water droplet symbol. ***E***, tPAC data analysis for the LFP recorded from CA1. For each electrode, the raw signal collected at 20 kHz [i, rewarded odorant (left), unrewarded odorant (right)] was filtered into different frequency bands (ii, θ 6–14 Hz). Hilbert transform was used to calculate the θ phase (iii) and the amplitude envelope of higher oscillations such as high γ (65-95 Hz; red line in v; blue line is the γ filtered LFP).

Male mice two to four months of age were anesthetized by brief exposure to isoflurane (2.5%) and subsequently anesthesia was maintained with an intraperitoneal injection of ketamine (100 mg/kg) and xylazine (10 mg/kg). The tetrode drive included one optical fiber ferrule for support of an EIB-8 board with two tetrodes with four polyamide-coated nichrome wires (diameter 12.5 μm; Sandvik, gold plated to an impedance of 0.2–0.4 MΩ). Tetrodes were connected, and the optic fiber ferrule was glued through an EIB-8 interface board (Neuralynx). Mice were implanted with two tetrode drives aimed at deep infralimbic mPFC (+1.94 mm anterior, +0.25 mm lateral, and −3.12 mm below bregma; Eleore et al., 2011) and the second tetrode drive at the right CA1 layer of the dorsal hippocampus (−3.16 mm posterior, +3.2 mm lateral, and −2 mm below bregma; Eleore et al., 2011; Fig. 1*A*). One ground screw was inserted 1 mm posterior from bregma and 1 mm lateral to the midline and sealed to the bone with dental acrylic. Mice were allowed to recover for one week before the initiation of the behavioral studies. All behavioral and LFP recording experiments were performed with 2.5- to 8-month-old mice that had undergone double tetrode drive implantation.

#### Go-no go behavioral task

**Figure 2.**
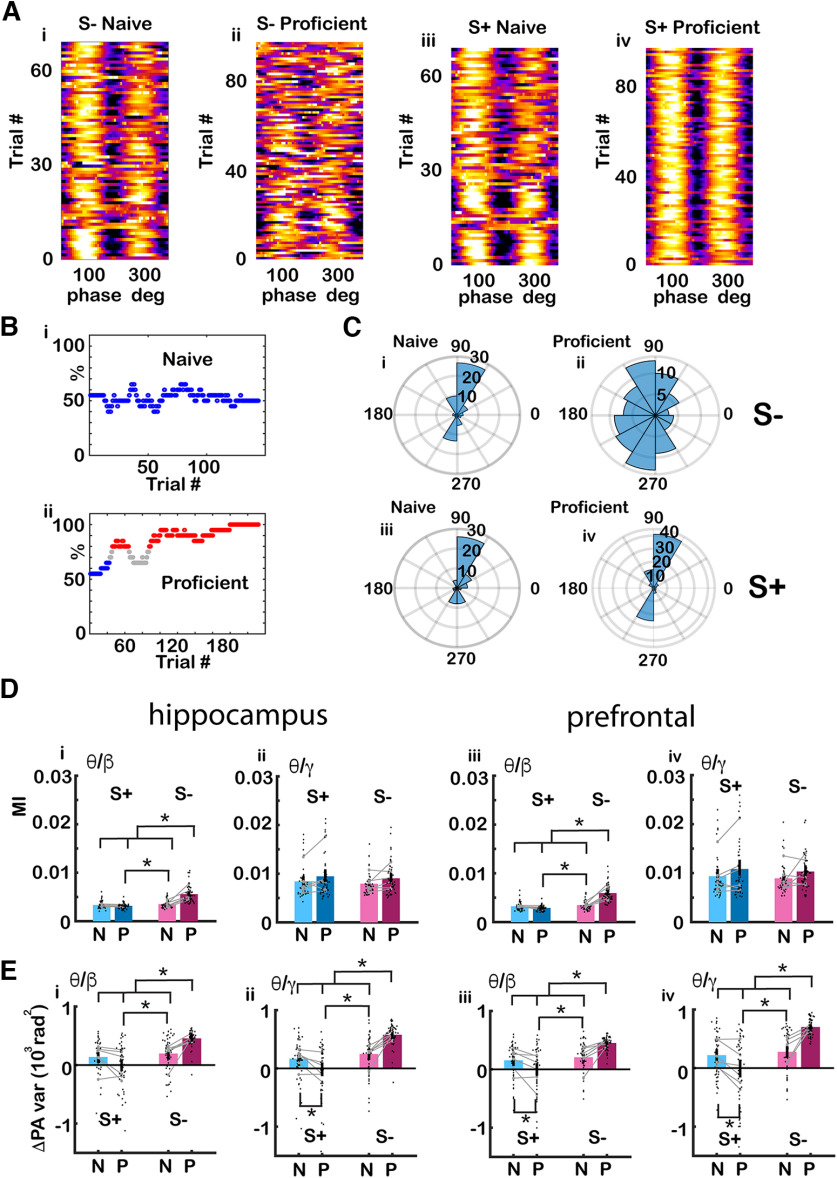
The peak angle variance of tPAC increased for the unrewarded odorant as the animal learned to discriminate odorants. ***A***, Pseudocolor plots showing the phase amplitude relationship for the unrewarded odorant S– (left) and rewarded odorant S+ (right) for two example sessions (naive and proficient) of the go-no go task computed for LFP recorded from CA1. As the animal became proficient differentiating between the rewarded and unrewarded odorants, tPAC peak angle appeared to become more variable for the unrewarded odorant (S–) compared with the rewarded odorant (S+). These pseudocolor plots are all in the same scale. ***B***, Behavioral performance for the two sessions. i, During the naive stage, the animal licked for the presentation of the rewarded and unrewarded odorants (performance ∼50%). ii, During the proficient stage (red points), the animal licked more frequently during the presentation of the rewarded odorant and refrained from licking during the presentation of the unrewarded odorant. Blue = percent correct <65%, red percent correct >80%. ***C***, Peak angle polar histograms for the examples in ***A***: (i) naive S–, (ii) proficient S–, (iii) naive S+, and (iv) proficient S+. The variance of the peak angle appears to increase for the S– in comparison to the S+ during the proficient stage. ***D***, Bar graphs showing the differences in MI for the odorant period (0.5–2.5 s after the odorant is diverted to the odor port) between S+ and S– for hippocampus (i) θ/β, (ii) θ/high γ and mPFC (iii) θ/β, (iv) θ/high γ. The black points are per mouse per odorant averages. The gray symbols and lines are per mouse averages. For the β tPAC for both the hippocampus and mPFC GLM found statistically significant differences for S+ versus S– and for the interaction between S+ versus S– and naive versus proficient (*p* < 0.001, 188 observations, 184 df, *F* statistic = 51–67.7, *p* < 0.001, 6 mice, 8 odor pairs; Extended Data Fig. 2-1). ***E***, Bar graphs showing differences for peak angle variance between for the odorant period (0.5–2.5 s after the odorant is diverted to the odor port) S+ and S– for hippocampus (i) for θ/β, (ii) θ/high γ and mPFC (iii), θ/β (iv) θ/high γ. The black points are per mouse per odorant averages. The gray symbols and lines are per mouse averages. For both β and γ tPAC and for both the hippocampus and mPFC GLM find statistically significant differences for S+ versus S– and for the interaction between S+ versus S– and naive versus proficient (*p* < 0.001, 188 observations, 184 df, *F* statistic = 19.5–26.4, *p* < 0.001, 6 mice, 8 odor pairs; Extended Data Fig. 2-1). Asterisks show significant *p* values (p< pFDR) for *post hoc* pairwise tests. Extended Data Figure 2-1 provides GLM and ANOVAN statistical analysis for the data in ***D*** and ***E***.

Water-restricted mice were required to enter an odor port and lick at the water spout to initiate the release of the odorants 1–1.5 s after the first lick (Fig. 1*B*,*C*; Losacco et al., 2020). Mice were required to lick at least once in four 0.5 s intervals during reinforced odorant delivery (S+) to obtain 10 μl of water. When exposed to the unreinforced odorant (S–), mice refrain from licking for 2 s. Licking was detected by closing a circuit between the licking spout and the grounded floor of the cage (Slotnick and Restrepo, 2005). The lick signal was recorded by the INTAN board in parallel with electrode recordings. The performance of the mice was assessed by calculating correct response to the S+ and S– odorants in 20 trial blocks where 10 S+ and 10 S– odorants were presented at random. Mice were first trained to discriminate between 1% isoamyl acetate (S+) and mineral oil (S–). Once the mice learned to discriminate between isoamyl acetate and mineral oil (percent correct > 80% in two blocks) the odorant pair was switched to the 1% acetophenone S+ and ethyl benzoate S– (APEB) odorant pair. Once the mice learned to discriminate between acetophenone and ethyl benzoate acetate the odor pair was reversed. Ethyl benzoate became the S+ and acetophenone the S– (EBAP). When the mice became proficient at the reversal, the odor pair was switched. The same pattern was followed for the rest of the odor pairs (see Table 2 for the list of all odorants used). All odorants were obtained from Sigma-Aldrich and were diluted in mineral oil at room temperature. Mice were trained until they performed at 80% correct or better in the last two blocks of 20 trials. Figure 2*B* shows an example of the percent correct odorant discrimination performance per trial. We did not find significant differences in the analysis between odorant pairs.

**Table 2:** Odorant pairs

Name	Rewarded odorant (S+)	Unrewarded odorant (S–)
APEB	1% acetophenone	1% ethyl benzoate
EBAP	1% ethyl benzoate	1% acetophenone
EAPA1	0.1% ethyl acetate	0.05% ethyl acetate + 0.05% propyl acetate
EAPA1	0.05% ethyl acetate + 0.05% propyl acetate	0.1% ethyl acetate
EAPA2	0.01% ethyl acetate	0.005% ethyl acetate + 0.005%propyl acetate
PAEA2	0.005% ethyl acetate + 0.005% propyl acetate	0.01% ethyl acetate
EAPA3	0.001% ethyl acetate	0.0005% ethyl acetate + 0.0005% propyl acetate
PAEA3	0.0005% ethyl acetate + 0.0005% propyl acetate	0.001% ethyl acetate

The odorants were diluted in odorless mineral oil.

### Double tetrode recordings

We followed procedures described by A Li et al. (2015). The mouse was recorded within the olfactometer chamber with dimensions of 11.6 × 9.7 × 9.4 cm. The EIB-8 boards that recorded signals from the tetrodes were connected to an INTAN RHD2132 16 channel amplifier/A/D converter that interfaced with an RHD2000 USB interface board. Extracellular potentials from the tetrodes were captured, filtered with either a 500 Hz or a 5-kHz low pass filter, and digitized at 20 kHz. Metadata for the behavioral events such as valve opening/closing times and odor identity were recorded through a digital output from the olfactometer. Licks detected by the olfactometer were recorded as an analog signal by the INTAN board.

### tPAC analysis

As described by Losacco et al. (2020), tPAC data were processed using the Hilbert transform using a method described by Tort et al. (2010). Briefly, the signal was bandpass filtered with a 20^th^ order Butterworth filter using MATLAB’s filtfilt function with zero phase shift to extract LFP in the low-frequency oscillation used for phase estimation and the high-frequency oscillation used for estimation of the amplitude of the envelope (Fig. 1*E*i,ii). The Hilbert transform established the θ (6–14 Hz) phase and the envelope for β (15–30 Hz) and high γ (65–95 Hz, referred to as γ; Fig. 1*E*iii,iv). To quantify the strength of tPAC, we calculated the modulation index (MI). If tPAC is nonexistent, MI = 0, meaning the mean amplitude is distributed uniformly over θ phases, and if tPAC is a δ function MI = 1. MI for signals measured in brain areas such as the hippocampus typically fall between 0 and 0.03 (Tort et al., 2010).

### tPRP analysis

As described by Losacco et al. (2020), the tPRP approach was developed using custom MATLAB code. tPAC was calculated following the approach used by Tort et al. (2010), as described in tPAC analysis and summarized in Figure 1. Peak and trough θ phases are defined as the phase for maxima and minima of the tPAC distribution measured for the S+ trial. A continuous Morlet wavelet transform was used to estimate the power for the high-frequency oscillations (Buonviso et al., 2003). tPRP was estimated as the power of the high-frequency oscillations measured at the peak or trough of tPAC. The MATLAB code used for data analysis has been deposited to GitHub. This analysis provides information on what information is gathered by a downstream observer using a phase window locked to peak or trough of the θ LFP of the rewarded odorant.

### Determination of divergence time from ztPRP and lick time courses

We computed a *p* value with a ranksum test to determine the time when ztPRP time courses and lick rates diverge between rewarded and unrewarded odorant trials in proficient animals. The divergence time was computed as the time point after odorant onset where the *p* value dropped below 0.005 for ≥1.2 s after addition of the odorant. Using this criterion on *p* value traces before odorant application resulted in finding a divergence because of fluctuations in the *p* values <5% of the cases.

### Imaginary Coherence (iCoherence) and Phase Locking Value (PLV) Analysis

We used two complementary methods to quantify coordinated neural activity by calculating imaginary coherence (iCoherence) and phase locking value (PLV), measures of coherence that are independent of volume conduction (Bastos and Schoffelen, 2016; Namburi, 2021; Nolte et al., 2004). We measured imaginary coherence following the method detailed in Nolte et al., 2004. The measure of imaginary coherence varies between -1 and 1 and indicates the strength and directionality of sustained phase differences between the two oscillations. Furthermore, we quantified phase-locking between the two LFPs by computing PLV following the procedure detailed by Lachaux et al. (1999) using MATLAB code generated by Praneeth Namburi (Namburi, 2021). Briefly, we compute the convolution of each LFP with a complex Gabor wavelet centered at frequency f and then we compute the PLV as the normalized absolute value of the sum of the exponential of the difference in phase multiplied by the imaginary number i. If the phase difference between the two LFPs varies little across the trials, PLV is close to 1; it is close to zero when the relationship between the phases varies randomly across trials.

### Statistical analysis

The statistical analysis was done as described by Losacco et al. (2020) using MATLAB code. Both tPAC and tPRP parameters were calculated separately per electrode (16 electrodes per mouse, 8 hippocampus, 8 mPFC) for all electrodes per mouse. Statistical significance for changes in measured parameters for multivariate factors such as the mouse genotype, naive versus proficient and S+ versus S–, and the interactions of these factors was estimated using generalized linear model (GLM) analysis, with *post hoc* tests for all data pairs corrected for multiple comparisons using false discovery rate (Curran-Everett, 2000). The *post hoc* comparisons between sets of data were performed either with a *t* test, or a ranksum test, depending on the result of an Anderson-Darling test of normality. Degrees of freedom and statistical significance have the same meaning in GLM as in analysis of variance and regression (Agresti, 2015). In addition, as a complementary assessment of significant differences (Halsey et al., 2015), we display 95% confidence intervals (CIs) shown in the figures as vertical black lines or shadow boundaries that was estimated by bootstrap analysis of the mean by sampling with replacement 1000 times. Furthermore, we ran an additional statistical analysis using a nested ANOVAN taking on account that each odorant pair is run for each mouse. The results of the GLM are more conservative than the nested ANOVAN. In the extended data for each figure, we provide the results for both the nested ANOVAN and the GLM analysis. In Results, we use the more conservative GLM test to make statistical decisions. Finally, to give visual information on the distribution of the data for the bar graphs the per odorant per mouse points are spread out along the *x*-axis according to their distribution.

### Linear discriminant analysis (LDA)

Decoding of contextual odorant identity from tPRP values was performed using LDA using MATLAB code as described by Losacco et al. (2020). LDA was trained by a leave one out procedure where the algorithm was trained with all trials but one, and the accuracy was assessed by predicting the contextual odorant identity for the trial that was left out. This was repeated for all trials and was performed separately for peak and trough tPRP, and for analysis where the identity of the odorants was shuffled. LDA was performed separately for the naive and proficient datasets on a per-mouse basis where the input was the tPRP recorded from 16 electrodes.

## Results

### Dual CA1-mPFC tetrode recording in mice undergoing the go-no go olfactory discrimination task

The goal of this study was to determine whether changes in coupled oscillations that occur in the OB as mice learn to discriminate odorants in a go-no go task are observed in downstream areas of the brain (mPFC and hippocampus). These downstream areas receive input originating from the OB and oscillations in the bulb are known to be coupled to the hippocampus and throughout the brain (Martin et al., 2007; Nguyen Chi et al., 2016; Tort et al., 2018). We also wanted to determine whether there was a difference between the CaMKIIα KO, CaMKIIα Hets, and the WT mice in tPAC that measures the amplitude of high bandwidth β and γ oscillations in different phases of slow θ oscillations. Furthermore, we determined whether the power of high bandwidth oscillations carry different information at the peak and trough high bandwidth amplitude phase of the θ oscillation for rewarded odorant trials. Evaluating tPRP allows for determining whether a downstream observer looking through these two θ phase windows receive different information on the stimulus. Lastly, we determined whether coordinated neural activity changed as the animal learned to discriminate odorants and we asked whether there was a difference in the relationship of oscillations between CA1 and mPFC between the different CaMKIIα genotypes.

In the go-no go odorant discrimination task, thirsty mice learned to lick on a spout in the presence of a rewarded odorant (S+) to obtain a water reward and refrained from licking in the presence of unrewarded odorant (S–) in a go-no go associative learning task (Losacco et al., 2020). The odorants were presented in pseudorandomized order in the go no-go task (Fig. 1*A–D*). The odorant pairs tested were different volatile compounds (Table 2) and we found no difference in any measurements between odorant pairs. Mice start the trial at will by licking on the lick port. The odorant is delivered at a random time 1–1.5 s after nose poke (the time course for the trial is shown in Fig. 1*C*). The mice learn to either lick a waterspout at least once during each 0.5-s segment in the 2-s response area to obtain a water reward for the rewarded odorant or refrain from licking for the unrewarded odorant. Mice refrain from licking during presentation of the unrewarded odorant because of the effort it takes to lick. Behavioral performance was termed naive or proficient when their performance estimated in a 20-trial window was below 65% for naive and above 80% for proficient. Mice were trained in the task for sessions of up to 200 trails. In the last session training ended after the animal achieved two 20-trial blocks of proficient performance; valence was reversed the next day or another odorant was tested. We recorded the LFP using two tetrodes (four electrodes per tetrode) implanted in the CA1 of the hippocampus and two tetrodes in ipsilateral mPFC, and we analyzed the data to determine whether the different genotypes differ in cross-frequency coupling for naive or proficient mice (Fig. 1*E*). The dataset is comprised of 747 recording sessions in 18 mice. The odorant pairs tested were different volatile compounds (or mixtures) whose nomenclature addresses the odorant names and the experimental set (e.g., APEB, see Table 2 for the nomenclature). Table 3 enumerates the total number of sessions per odorant pair, mouse, and experiment.

**Table 3:** Number of sessions and number of mice per odorant pair (**Figs. 1-13)**

Odorant pair	Number of mice	Total sessions
1% acetophenone vs 1% ethyl benzoate	6 WT	22
	7 Het	22
	5 KO	11
1% ethyl benzoate vs 1% acetophenone	6 WT	22
	7 Het	55
	5 KO	29
0.1% ethyl acetate vs 0.05% ethyl acetate + 0.05% propyl acetate	6 WT	15
	7 Het	23
	5 KO	17
0.05% ethyl acetate + 0.05% propyl acetate vs 0.1% ethyl acetate	6 WT	17
	7 Het	35
	5 KO	37
0.01% ethyl acetate vs 0.005% ethyl acetate + 0.005% propyl acetate	6 WT	23
	7 Het	52
	4 KO	24
0.005% ethyl acetate + 0.005% propyl acetate vs 0.01% ethyl acetate	6 WT	31
	7 Het	49
	4 KO	21
0.001% ethyl acetate vs 0.0005% ethyl acetate + 0.0005% propyl acetate	6 WT	60
	7 Het	64
	3 KO	16
0.0005% ethyl acetate + 0.0005% propyl acetate vs 0.001% ethyl acetate	6 WT	34
	7 Het	57
	3 KO	13

Note: We used a total of 18 mice: 6 WT, 7 Het, and 5 KO, but, as shown in column 2, not all mice were tested with all odorant pairs.

We performed tPAC analysis of the LFP recorded in the go no-go behavioral task. tPAC analysis is a cross-frequency coupling analysis to determine whether high-frequency oscillation bursts take place at specific phases of low-frequency θ oscillations (Tort et al., 2010). tPAC has been reported in brain areas such as the OB, hippocampus and PFC (Belluscio et al., 2012; Kaplan et al., 2014; Rojas-Líbano et al., 2014; Colgin, 2015; Scheffer-Teixeira and Tort, 2016; Losacco et al., 2020). Figure 1*E*i–iv shows an example of high γ tPAC for the LFP recorded in CA1. Figure 1*E*i shows the extracellular LFP sampled for hippocampus at 20 kHz and filtered between 1–750 Hz. The raw signal (Fig. 1*E*i) was filtered with a 20^th^ order Butterworth filter into different LFP frequency bands [Fig. 1*E*ii–iv; θ, 6–14 Hz, adapted from Nguyen Chi et al. (2016) high γ, 65–95 Hz]. We used tPAC analysis (Tort et al., 2010) to evaluate the degree of coupling of the amplitude of the envelope of the β or high γ LFP on the phase of the θ LFP. Figure 1*E*iii shows the θ phase and in Figure 1*E*iv shows the envelope for the amplitude of the high γ LFP, both calculated with the Hilbert transform as detailed by Tort et al. (2010). Figure 1*E*ii,iv shows that the filtered high γ LFP changes amplitude in manner that appears coordinated with the θ phase.

### The peak angle variance of tPAC increased for the unrewarded odorant as the animals became proficient differentiating between odorants

We proceeded to ask whether the strength of tPAC, quantified by the MI, changes as the animal learns to differentiate odorants in the go-no go task. MI is a measure of how localized high-frequency oscillation is within the phase of θ oscillations (Tort et al., 2010). Figure 2*A–C* illustrate an example of high γ tPAC recorded in CA1 for S+ and S– odorant trials in two sessions for naive and proficient mice. The phase amplitude plots for the trials are shown in pseudocolor in Figure 2*A*, for a WT mouse during the naive (Fig. 2*A*i–iii) and proficient (Fig. 2*A*ii–iv) stages and the percent correct as a function of trial number is shown for the two sessions in Figure 2*B*. In this example, there appears to be an increase in the strength of tPAC when the animal becomes proficient differentiating between the odorants. Furthermore, there was an increase in peak angle variance for the unrewarded odorant as shown by the peak angle polar histograms in Figure 2*C*i,ii. Figure 2*C*i shows that for the unrewarded odorant the peak angle was near 90° during the naive stage, while Figure 2*C*ii shows the peak angle for the unrewarded odorant fluctuated widely during the proficient stage. This is in contrast with the rewarded odorant for which the peak angle remained near 90° during naive (Fig. 2*C*iii) and proficient stages (Fig. 2*C*iv).

Figure 2*D* shows the differences in MI computed per mouse, per odorant pair between S+ and S– for naive and proficient mice for tPAC for hippocampus (Fig. 2*D*i) β, (Fig. 2*D*ii) high γ and mPFC (Fig. 2*D*iii) β, (Fig. 2*D*iv) high γ. For the β tPAC MI for both the hippocampus and mPFC a GLM analysis found statistically significant differences between S+ versus S– and the interaction between S+ versus S– and naive versus proficient (*p* < 0.001, 188 observations, 184 df, *F* statistic = 51–67.7, *p* < 0.001, 6 mice, 8 odor pairs; Extended Data Fig. 2-1). GLM does not find significant differences for MI for high γ tPAC (*p* > 0.05). Figure 2*E* shows differences for peak angle variance between S+ and S– for naive and proficient mice for hippocampus tPAC (Fig. 2*E*i) for β and (Fig. 2*E*ii) high γ and for mPFC tPAC for (Fig. 2*E*iii) β and (Fig. 2*E*iv) high γ. For both β and γ tPAC and for both the hippocampus and mPFC GLM found statistically significant differences between S+ versus S– and the interaction between S+ versus S– and naive versus proficient (*p* < 0.001, 188 observations, 184 df, *F* statistic = 19.5–26.4, *p* < 0.001, 6 mice, 8 odor pairs; Extended Data Fig. 2-1) and for naive versus proficient (*p* < 0.05). Asterisks in all figures show pairwise statistical significance (*t* test or ranksum test, *p*<pFDR, *p* value for significance corrected for multiple comparisons using the false discovery rate; Curran-Everett, 2000).

Overall, we found an increase in peak angle variance for the unrewarded odorant as the animal became proficient. We also found small, but significant changes in the strength of β tPAC that differed between the S+ and S– odorants as the animal learned to discriminate odorants.

### The odorant-elicited change in the θ phase-referenced β and γ power became negative for the unrewarded odorant as the mice became proficient discriminating between odorants

**Figure 3.**
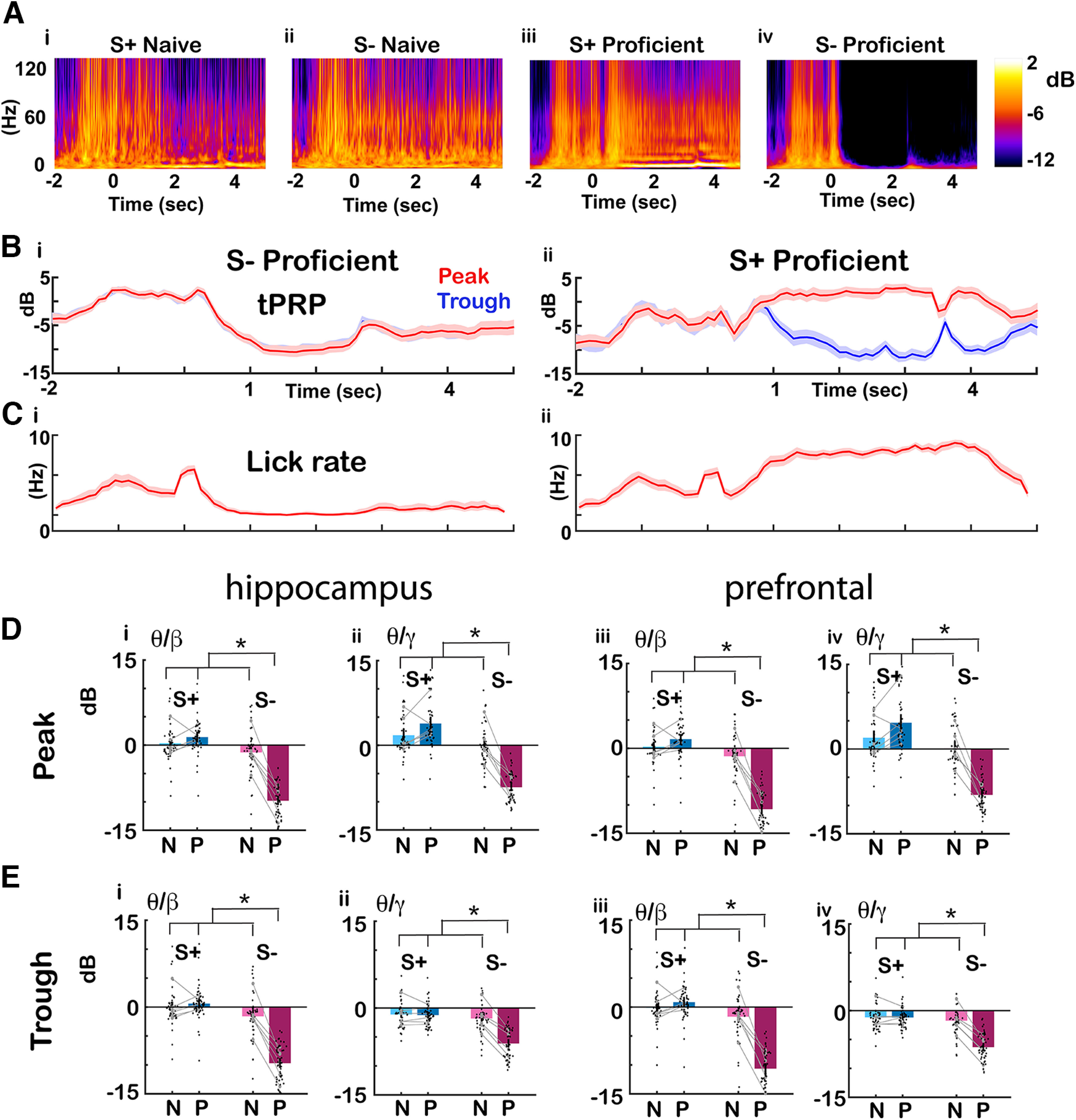
Changes in phase-referenced power as the animal became proficient. ***A***, Examples of the time course per trial for the average wavelet broadband LFP spectrogram for (i) S+ naive (30 trials), (ii) S– naive (27 trials), (iii) S+ proficient (84 trials), (iv) S– proficient (84 trials). Pseudocolor scale for LFP power is shown in dB. The LFP was recorded from CA1. ***B***, Average γ wavelet power referenced to θ peak and through for the same trials as shown in ***A*** (i) S– proficient, (ii) S+ proficient, red: peak, blue: through, shadow: confidence interval. ***C***, Lick rate for the same trials (i) S– proficient, (ii) S+ proficient. ***D***, ***E***, Summary bar graphs showing that peak/trough tPRP for the odorant period (0.5–2.5 s after the odorant is diverted to the odor port) decreases for S– as the mice become proficient. Black points are per mouse per odorant averages. The gray symbols and lines are per mouse averages. ***D***, Peak. ***E***, Through. (i) θ/β and (ii) θ/γ hippocampus, (iii) θ/β and (iv) θ/γ PFC. GLM found statistically significant differences for S+ versus S– and for the interaction between S+ versus S– and naive versus proficient (*p* < 0.001, 376 observations, 368 df, *F* statistic = 72.7–103, *p* < 0.001, 6 mice, 8 odor pairs; Extended Data Fig. 3-1). Asterisks show significant *p* values (*p*<pFDR) for *post hoc* pairwise tests. Extended Data Figure 3-1 provides GLM and ANOVAN statistical analysis for the data in ***D***, ***E***.

Wavelet power referenced to θ phase (peak or trough) was determined to evaluate whether it changes as the animal learns. This analysis is referred to as θ phase-referenced power (tPRP; Losacco et al., 2020). Figure 3*A* shows examples for CA1 of the time course during the trial for the average wavelet broadband LFP spectrograms for 30 trials during naive S+ (Fig. 3*A*i), 27 trials during naive S– (Fig. 3*A*ii) 84 trials during proficient S+ (Fig. 3*A*iii) and 84 trials during proficient S– (Fig. 3*A*iv). After odorant onset, there was an increase in broadband power for the S+ odorant and a decrease in power for the S– odorant as the animal became proficient.

Figure 3*B* shows for this example for LFP recorded in CA1 the time course during the trial for the average high γ tPRP referenced to the peak (red) or trough (blue) of θ for the proficient trials in Figure 3*A*. For the rewarded (S+) odorant the power increased for the peak and decreased for trough after addition of the odorant while for the unrewarded odorant both peak and trough tPRPs decreased. Figure 3*C* shows that, as expected, there was an increase in the lick rate for the rewarded odorant and a decrease for the unrewarded odorant. Finally, Figure 3*D*,*E* shows a per mouse per odorant pair analysis that indicated that tPRP became negative for S– as the mice became proficient. Figure 3*E*, peak; Figure 3*F*, through, (i) β and (ii) γ hippocampus; (iii) β and (iv) γ mPFC. GLM found statistically significant differences for tPRP between S+ versus S– and the interaction between S+ versus S– and naive versus proficient (*p* < 0.001, 376 observations, 368 df, *F* statistic = 72.7–103, *p* < 0.001, 6 mice, 8 odor pairs; Extended Data Fig. 3-1).

**Figure 4.**
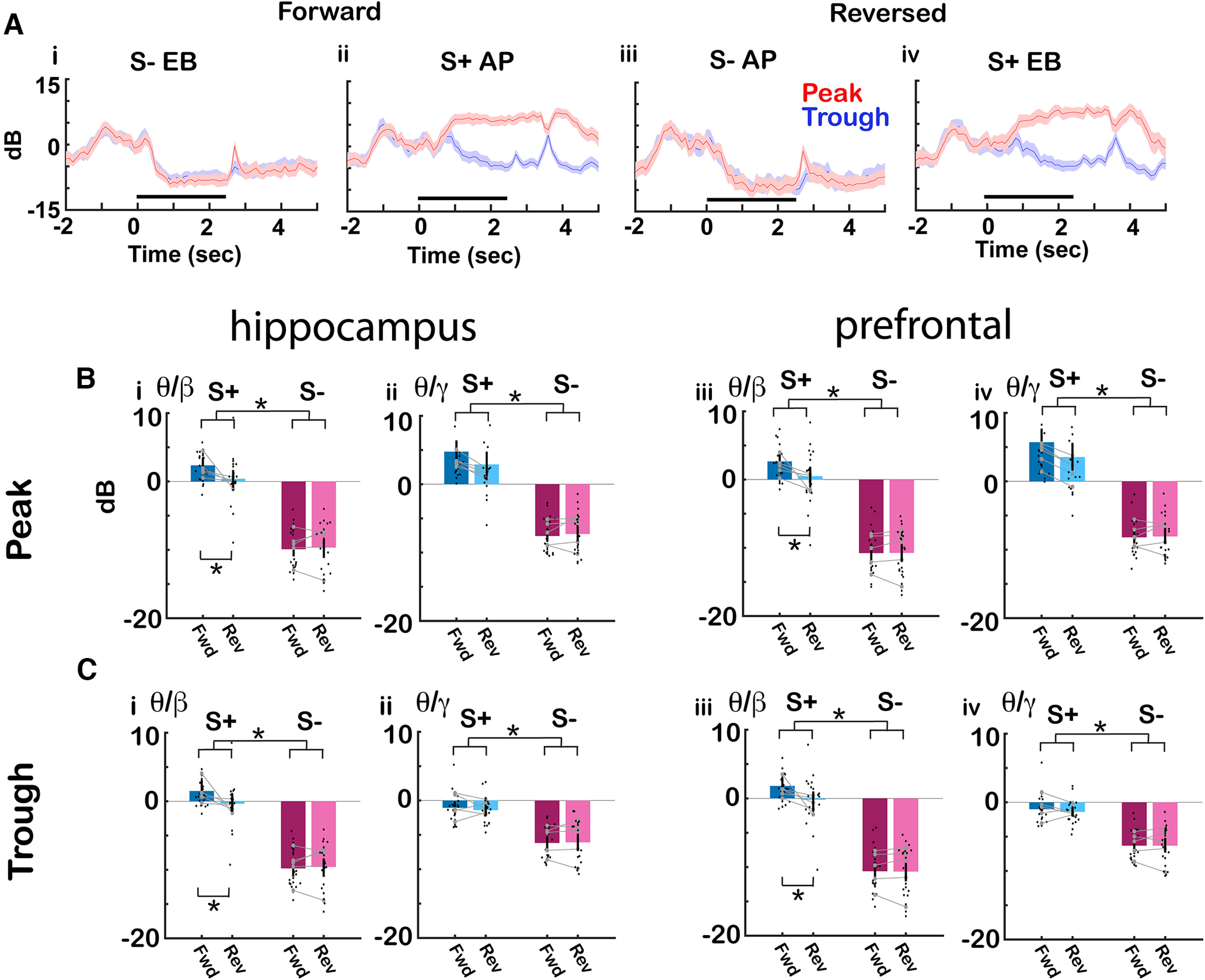
Effect of reversal of odorant valence on the phase-referenced power. ***A***, Average γ wavelet power referenced to θ peak and through for the same trials when the mouse was proficient for the CA1 LFP example shown in Figure 3*A*. The γ tPRP is normalized by subtracting the power before odorant application. i, ii, Forward: (i) S– (ethyl benzoate, EB), (ii) S+ (acetophenone, AP). iii, iv, Reversed: (iii) S– (acetophenone, AP), (iv) S+ (ethyl benzoate, EB), red: peak, blue: through, shadow; confidence interval. ***B***, ***C***, Summary bar graphs showing that the average peak tPRP for the odorant period (0.5–2.5 s after the odorant is diverted to the odor port) decreases for S– on odorant application regardless of the identity of the odorant. Black points are per mouse per odorant averages. The gray symbols and lines are per mouse averages. ***B***, Peak. ***C***, Through. (i) θ/β and (ii) θ/γ hippocampus, (iii) θ/β and (iv) θ/γ PFC. For β tPRP GLM found statistically significant differences for forward versus reverse (*p* < 0.05) and S+ versus S– (*p* < 0.001, 188 observations, 180 df, *F* statistic = 87, *p* < 0.001, 6 mice, 8 odor pairs; Extended Data Fig. 4-1). For γ tPRP, GLM found statistically significant differences for S+ versus S– and peak versus trough (*p* < 0.001, 188 observations, 180 df, *F* statistic = 61.8, *p* < 0.001, 6 mice, 8 odor pairs; Extended Data Fig. 4-1). Asterisks show significant *p* values (*p*<pFDR) for *post hoc* pairwise tests. Extended Data Figure 4-1 provides GLM and ANOVAN statistical analysis for the data in ***B*** and ***C***.

In addition, we asked whether odorant-induced changes in peak tPRP changed when odorant valence was reversed. Figure 4*A*i,ii shows an example for CA1 LFP of the time course for the peak (red) and trough (blue) high γ tPRP when the mouse was proficient for a forward session where the rewarded odorant (S+) was acetophenone (AP) and the unrewarded odorant (S–) was ethyl benzoate (EB). Figure 4*A*iii,iv shows that when the valence of the odorant was reversed (AP was S– and EB was S+) the response to EB resembled the response to AP in the forward sessions (compare Fig. 4*A*ii and iv) indicating that the response is a response to the contextual identity of the odorant (is it rewarded?) as opposed to the chemical identity of the odorant. Figure 4*B*,*C* shows a summary bar graph analysis per mouse per odorant pair of all reversal experiments indicating that for the proficient mouse the average peak and trough tPRP decreases for the unrewarded S– odorant regardless of the identity of the odorant. For high γ tPRP GLM found statistically significant differences for S+ versus S– and peak versus trough (*p* < 0.001, 188 observations, 180 df, *F* statistic = 61.8, *p* < 0.001, 6 mice, 8 odor pairs; Extended Data Fig. 4-1) and does not find a difference between forward and reverse sessions indicating that indeed the high γ tPRP responds to the contextual odorant identity. For β tPRP GLM found a statistically significant difference for S+ versus S– (*p* < 0.001, 188 observations, 180 df, *F* statistic = 87, *p* < 0.001, 6 mice, 8 odor pairs; Extended Data Fig. 4-1) and interestingly, GLM does find a small statistically significant differences between forward versus reverse (*p* < 0.05) for β tPRP. Although this is a relatively small difference, this indicates that β tPRP is not exclusively responsive to contextual odorant identity and that these brain regions may also encode information on the chemical identity of the odorant.

### The accuracy for decoding the contextual identity of the odorants from θ phase-referenced power increased when the mice became proficient

We proceeded to determine whether we could decode contextual odorant identity from tPRP. Decoding was performed using a LDA to set a decision boundary hyperplane between binary stimulus classes (S+ vs S–; Vizcay et al., 2015). LDA was trained with tPRP from each electrode for each mouse (8 electrodes in the hippocampus and 8 electrodes in the mPFC per mouse) for all trials except one (the training dataset) and then the tPRP from the missing trial (test data) was classified as S+ or S–. This training was performed separately at both naive and proficient learning stages. As a control we shuffled the identity of trials in the training set.

**Figure 5.**
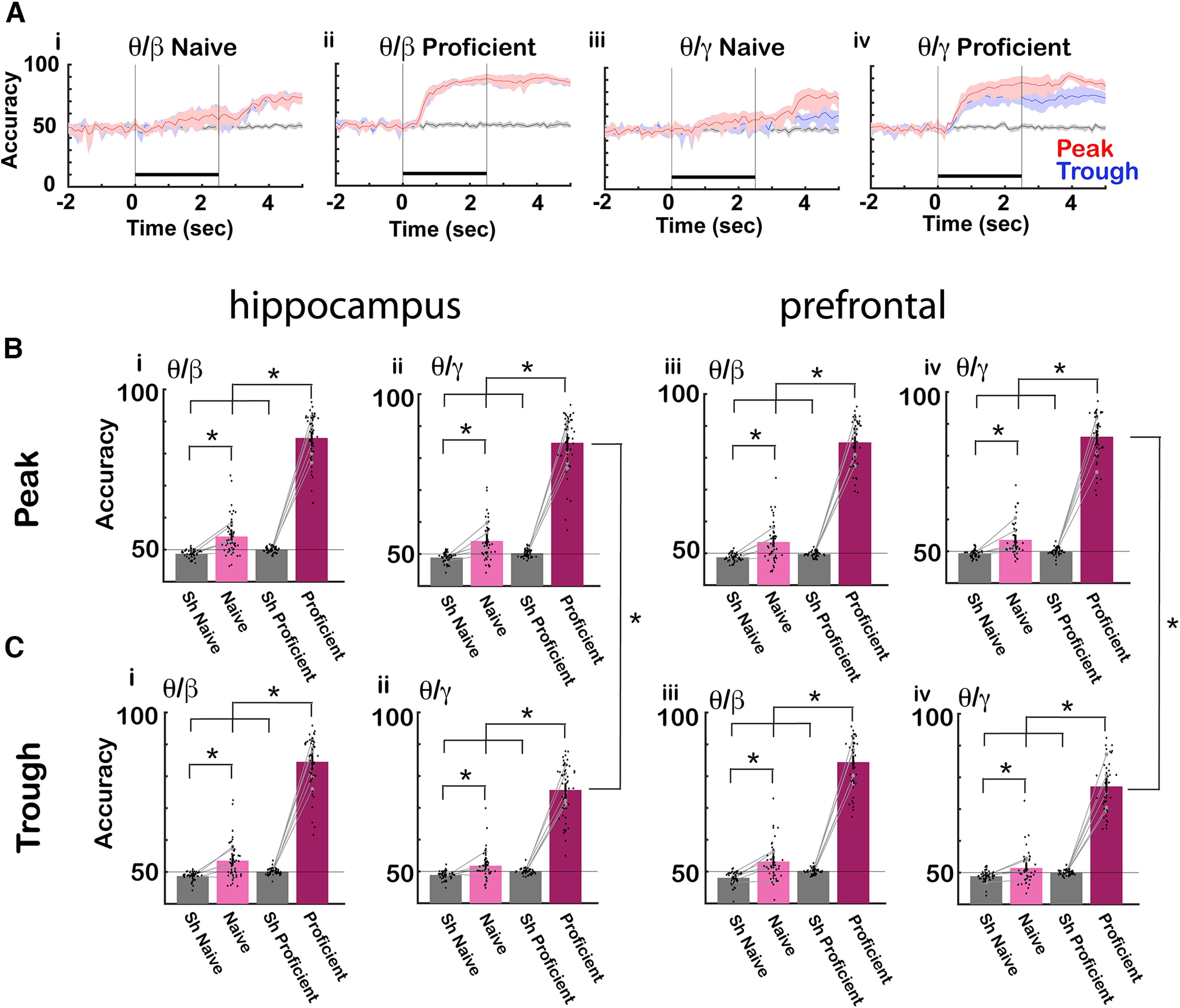
LDA for decoding the contextual odorant identity from tPRP. ***A***, Example for one mouse for the time course for the accuracy of odorant identity decoding by a LDA algorithm trained using tPRP calculated from CA1 LFP for the EAPA odor pair (i) naive θ/β, θ (ii) proficient θ/β, (iii) naive θ/γ, (iv) proficient θ/γ red: peak, blue: through, black: shuffled, shadow: confidence interval, black bar: odorant application. ***B***, ***C***, Bar graphs showing the differences in decoding accuracy between shuffled, naive, and proficient. ***B***, Accuracy for peak tPRP for (i) θ/β in the hippocampus, (ii) θ/γ in the hippocampus, (iii) θ/β in mPFC, (iv) θ/γ in mPFC. ***C***, Accuracy for through for (i) θ/β in the hippocampus, (ii) θ/γ in the hippocampus, (iii) θ/β in mPFC, (iv) θ/γ mPFC. The bars show the average accuracy, and the points are the accuracy per mouse per odor pair. The vertical bars show the confidence interval. The gray symbols and lines are per mouse averages. For β and γ tPRP for both prefrontal and hippocampus LDA, GLM found statistically significant differences for naive versus proficient and shuffled versus proficient (*p* < 0.001, 380 observations, 372 df, *F* statistic = 355–494, *p* < 0.001, 6 mice, 8 odor pairs; Extended Data Fig. 5-1). For γ tPRP for both prefrontal and hippocampus LDA, GLM found statistically significant differences between peak and trough (*p* < 0.05, 380 observations, 372 df, *F* statistic = 355–494, *p* < 0.001, 6 mice, 8 odor pairs; Extended Data Fig. 5-1). Asterisks show significant *p* values (*p*<pFDR) for *post hoc* pairwise tests. Extended Data Figure 5-1 provides GLM and ANOVAN statistical analysis for the data in ***B*** and ***C***.

Figure 5*A* shows an example of the time course during the trial for the decoding accuracy for one mouse for the LDA trained using CA1 tPRP for the EAPA odor pair for (Fig. 5*A*i) naive stage β, (Fig. 5*A*ii) proficient stage β, (Fig. 5*A*iii) naive stage γ, (Fig. 5*A*iv) proficient stage γ. For the naive animal decoding accuracy increased slowly after the addition of the odorant, and increased further after the animal received the reward (Fig. 5*A*i,iii). When the animal became proficient decoding accuracy increased rapidly beyond 80% after addition of the odorant (Fig. 5*A*ii,iv). For high γ tPRP the accuracy was higher for the proficient animal for peak-referenced tPRP compared with trough-referenced tPRP (Fig. 5*A*iv). Figure 5*B*,*C* shows mean bar graphs for the mean accuracy for decoding contextual odorant identity calculated for the last second of the odorant epoch (1.5–2.5 s after diverting the odorant toward the mouse) for shuffled, naive, and proficient. Figure 5*B* shows the accuracy for the peak tPRP and Figure 5*C* shows the accuracy for trough tPRP. For all conditions the accuracy is significantly higher for the proficient stage compared with naive or shuffled. GLM analysis found for both CA1 and mPFC statistically significant differences between naive versus proficient and shuffled versus proficient for both β and γ tPRP (*p* < 0.001, 380 observations, 372 df, *F* statistic = 355–494, *p* < 0.001, 6 mice, 8 odor pairs; Extended Data Fig. 5-1) and for γ tPRP GLM found statistically significant differences between peak and trough (*p* < 0.05, 380 observations, 372 df, *F* statistic = 355–494, *p* < 0.001, 6 mice, 8 odor pairs; Extended Data Fig. 5-1).

### θ Phase-referenced power diverges between rewarded and unrewarded trials before divergence of lick behavior

**Figure 6.**
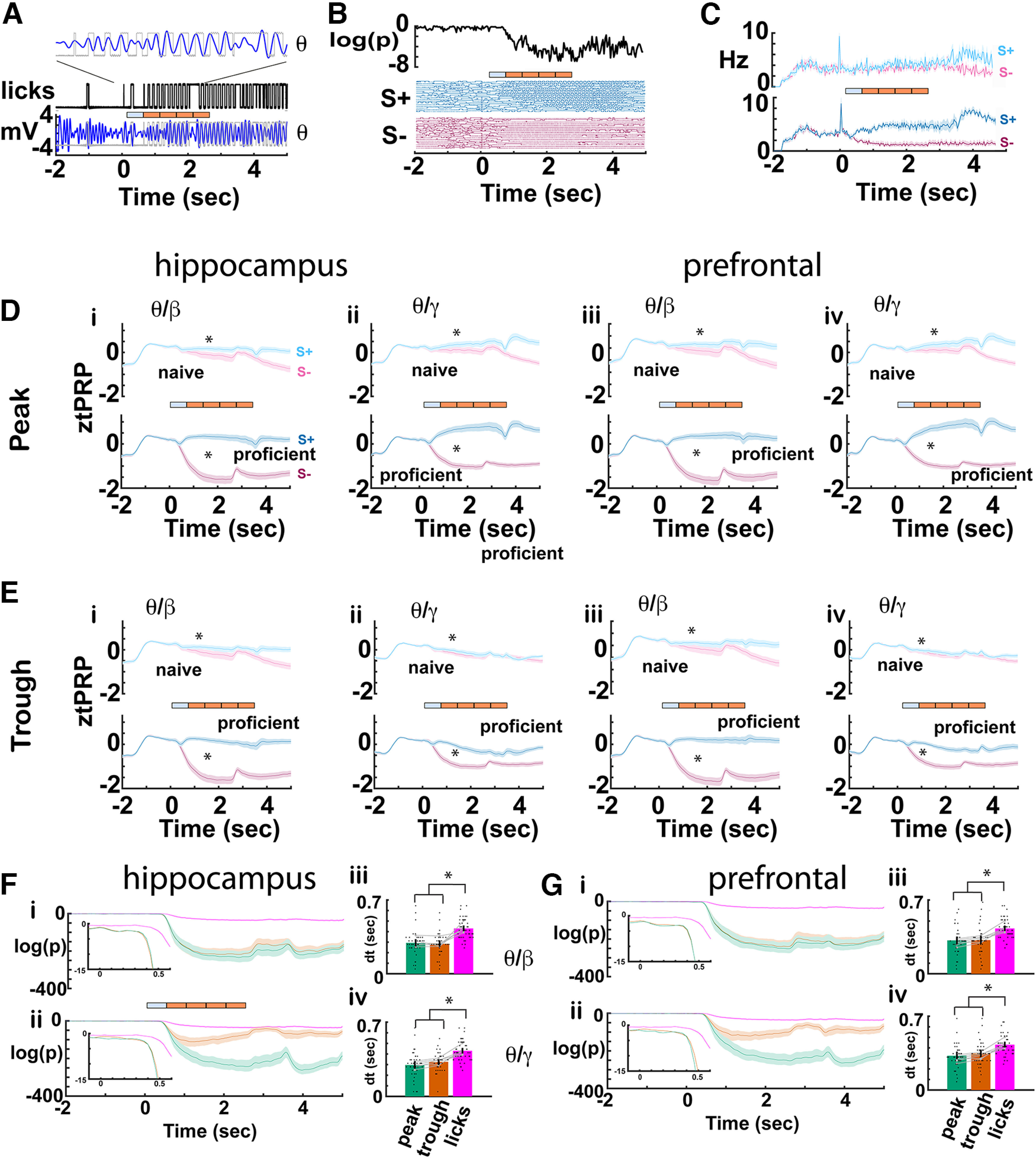
Comparison of divergence between rewarded and unrewarded trial time courses for lick rate and tPRP. ***A***, Example illustrating the relationship between licks and θ CA1 LFP for a Hit trial. The lick signal is binary with licks represented by an increase in voltage. The bar shows the 2.5-s odorant period divided in 0.5-s response areas. The mouse must lick at least once in each of the last four response areas to obtain a reward. ***B***, Lick time course for a proficient mouse for 18 S+ and 18 S– trials (bottom) and for the *p* value for the ranksum test comparing the S+ and S– binary lick traces (calculated in 33-ms time bins). ***C***, Average lick rate time course for the APEB odorant pair (±CI) for naive and proficient mice for the rewarded (S+) and unrewarded (S–) odorants (average of 5 mice). ***D***, ***E***, Time course for the average tPRP. The tPRP is z normalized by subtracting the mean tPRPfrom −2–0 s and dividing by the standard deviation in this period. The bounded line shows the average ztPRP bounded by the CI calculated from ztPRP traces per odorant per mouse (6 mice, 8 odor pairs). D is the peak ztPRP and E is the trough ztPRP. i, hippocampus θ/β, ii, hippocampus θ/γ, iii, mPFC θ/β and iv, mPFC θ/γ. Cyan is S+ and magenta is S–. GLM for the average β ztPRP between 0.5 and 2.5 s found statistically significant differences for both CA1 and mPFC for both naive versus proficient, rewarded versus unrewarded odorant and for the interaction between naive versus proficient and rewarded versus unrewarded odorant (*p* < 0.001, 376 observations, 368 df, *F* statistic = 110–104, *p* < 0.001, 6 mice, 8 odor pairs; Extended Data Fig. 6-1). GLM for the average γ ztPRP between 0.5 and 2.5 s found statistically significant differences for both CA1 and mPFC for both naive versus proficient (*p* < 0.001), rewarded versus unrewarded odorant (*p* < 0.001) and for the interactions between naive versus proficient and rewarded versus unrewarded odorant (*p* < 0.001), naive versus proficient and peak versus trough (*p* < 0.05) and rewarded versus unrewarded odorant and peak versus trough (*p* < 0.001, 376 observations, 368 df, *F* statistic = 88.9-94.5, *p* < 0.001, 6 mice, 8 odor pairs; Extended Data Fig. 6-1). Asterisks denote significant difference between rewarded and unrewarded odorants determined *post hoc* with *t* or ranksum tests (*p*<pFDR). ***F***, ***G***, i, ii, Time courses for the *p* value calculated with a ranksum test of the difference between rewarded and unrewarded trials for the ztPRP or the licks. The ranksum test was calculated in time bins of 33 ms. Shown are bounded lines of the average *p* value for ztPRP or licks (±CI) calculated per odorant per mouse. ***F*** (i) β CA1, ***F*** (ii) γ CA1, ***G*** (i) β mPFC, ***G*** (ii) γ mPFC. iii, iv, Divergence times between rewarded and unrewarded ztPRP and licks calculated from the ranksum *p* value time courses (see Materials and Methods). A GLM for the divergence times found statistically significant differences for both CA1 and mPFC between peak and licks and trough and licks (*p* < 0.001, 133 observations, 130 df, *F* statistic = 11.6–21, *p* < 0.001, 6 mice, 8 odor pairs; Extended Data Fig. 6-1). Asterisks denote significant differences determined *post hoc* with *t* or ranksum tests (*p* < pFDR). Extended Data Figure 6-1 provides GLM and ANOVAN statistical analysis for the data in ***D–G***.

θ Oscillations in mPFC are phase locked with licks in rats consuming liquid sucrose rewards and this θ range activity has been postulated to encode for the value of consumed fluids (Amarante et al., 2017; Amarante and Laubach, 2021). We proceeded to analyze the relationship between the tPRP time course in CA1 and mPFC and the time course for licks in the go-no go task. Figure 6*A* shows that licks are phase locked to the θ LFP for an example of licks aligned with θ LFP recorded from CA1 for a hit trial. Figure 6*B* shows the lick traces for the rewarded and unrewarded odorants for a proficient animal engaged in the go-no go task with the APEB odorant pair. For the rewarded odorant the animal licks continuously for several seconds after odorant application while for the unrewarded odorant the animal stops licking shortly after the odorant is delivered. The top panel shows that the *p* value calculated using a ranksum test of the difference in binary lick recordings between S+ and S– trials decreases sharply shortly after addition of the odorant reflecting divergence in lick behavior between the rewarded and unrewarded odorants. Figure 6*C* shows the mean lick rate for five mice undergoing the go-no go task for the APEB odorant pair.

In order to compare the divergence of lick behavior with tPRP we compared the time course for the decrease of *p* value estimating divergence between S+ and S– for licks versus tPRP. Figure 6*D*,*E* shows the time course for the z normalized peak and trough tPRP (ztPRP) for naive and proficient animals for the different bandwidths for CA1 (Fig. 6*D*i,ii, *E*i,ii) and mPFC (Fig. 6*D*iii,iv, *E*iii,iv). For proficient mice ztPRP diverged between rewarded and unrewarded odorant shortly after odor onset. Consistent with the results in Figure 3, a GLM analysis for the average β ztPRP between 0.5 and 2.5 s found statistically significant differences for both CA1 and mPFC for naive versus proficient, rewarded versus unrewarded odorant and for the interaction between naive versus proficient and rewarded versus unrewarded odorant (*p* < 0.001, 376 observations, 368 df, *F* statistic = 110–104, *p* < 0.001, 6 mice, 8 odor pairs; Extended Data Fig. 6-1). GLM for the average γ ztPRP between 0.5 and 2.5 s found statistically significant differences for both CA1 and mPFC for naive versus proficient (*p* < 0.001), rewarded versus unrewarded odorant (*p* < 0.001) and for the interactions between naive versus proficient and rewarded versus unrewarded odorant (*p* < 0.001), naive versus proficient and peak versus trough (*p* < 0.05), and rewarded versus unrewarded odorant and peak versus trough (*p* < 0.001, 376 observations, 368 df, *F* statistic = 88.9–94.5, *p* < 0.001, 6 mice, 8 odor pairs; Extended Data Fig. 6-1).

To estimate the time for divergence of ztPRP between rewarded and unrewarded trials in proficient mice, we calculated the *p* value for a two tailed *t* test for ztPRP for each mouse for each odorant pair and compared it to the time course for the *p* value for divergence of lick behavior. As shown for CA1 and mPFC in Figure 6*F*i,ii, *G*i,ii, there was a sharp decline in the *p* values shortly after addition of the odorant and the decrease in *p* value took place at earlier times of ztPRP compared with licks. Divergence time was computed as the time point after odorant onset where the *p* value dropped below 0.005 for ≥1.2 s after addition of the odorant. Figure 6*F*iii,iv, *G*iii,iv shows the time for divergence for peak and trough ztPRP compared with lick behavior. A GLM found statistically significant differences for both CA1 and mPFC for divergence time between peak ztPRP and licks and trough ztPRP and licks (*p* < 0.001, 133 observations, 130 df, *F* statistic = 11.6–21, *p* < 0.001, 6 mice, 8 odor pairs; Extended Data Fig. 6-1). Asterisks denote significant differences determined *post hoc* with *t* or ranksum tests (*p*<pFDR).

### The time for divergence between rewarded and unrewarded trials differs between pre-lick-referenced and post-lick-referenced tPRP

**Figure 7.**
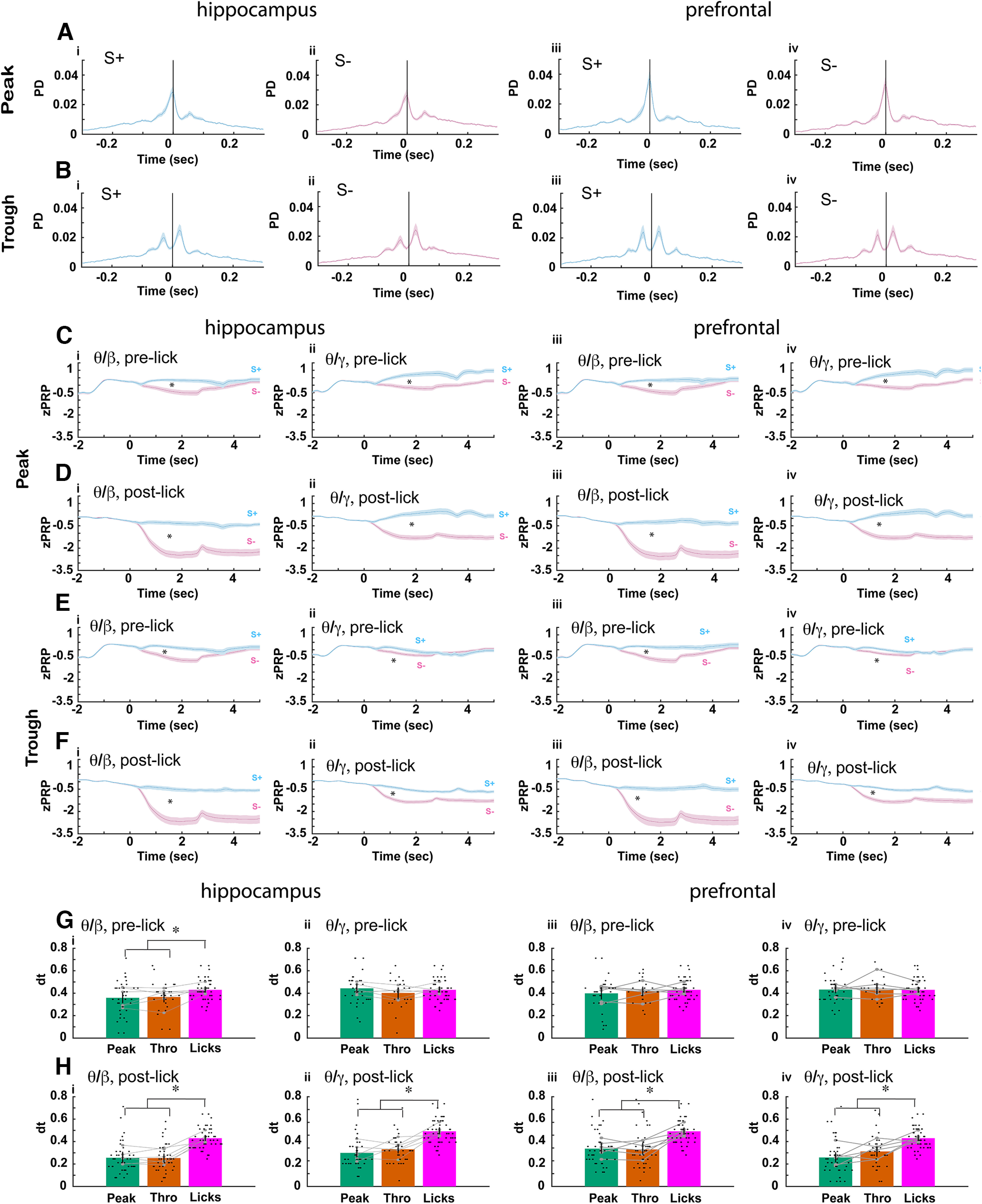
Lick-referenced tPRP for proficient mice. ***A–D***, Plots of the probability density (PD) for the time of peaks (***A***) or troughs (***B***) of θ oscillations referenced to the time for adjacent lick onset shown for CA1 (i, ii) and mPFC (iii, iv) for S+ (***A***i, ***B***i, ***A***iii, ***B***iii) and S– (***A***ii, ***B***ii, ***A***iv, ***B***iv). The plots show a bounded line (average ± CI) calculated from all the per mouse per odorant data. ***C–F***, Time course for the average lick-referenced tPRP. ***C*** and ***D*** are for peak lick-referenced tPRP and ***E*** and ***F*** are for trough lick-referenced tPRP. ***C*** and ***E*** are pre-lick lick-referenced tPRP calculated for peaks and troughs that took place before the reference lick, and ***D*** and ***F*** are post-lick lick-referenced tPRP calculated for peaks and troughs that took place after the reference lick. The tPRP is z normalized by subtracting the mean tPRPfrom −2–0 s and dividing by the standard deviation in this period. The bounded line shows the average ztPRP bounded by the CI calculated from ztPRP traces per odorant per mouse (6 mice, 8 odor pairs). i, hippocampus θ/β, ii, hippocampus θ/γ, iii, mPFC θ/β, and iv, mPFC θ/γ. Cyan is S+ and magenta is S–. GLM for the average β or γ ztPRP between 0.5 and 2.5 s found statistically significant differences for both CA1 and mPFC for naive versus proficient (naive are not shown in the figure), rewarded versus unrewarded odorant and pre-lick versus post-lick (*p* < 0.001, 752 observations, 736 df, *F* statistic = 91–123, *p* < 0.001, 6 mice, 8 odor pairs; Extended Data Fig. 7-1). Asterisks denote significant difference between rewarded and unrewarded odorants determined *post hoc* with *t* or ranksum tests (*p* < pFDR). ***G***, ***H***, Divergence times between rewarded and unrewarded lick-referenced ztPRP and licks calculated from the *p* value time courses (see Materials and Methods). ***G*** is pre-lick-referenced ztPRP, and ***H*** is post-lick-referenced ztPRP. i and iii are for β lick-referenced ztPRP, and ii and iv are for γ lick-referenced ztPRP. A GLM for the divergence times for pre-lick tPRP found no statistically significant differences between licks and either peak or trough for all bandwidths for mPFC (*p* > 0.05, 100 observations, 97 df, *F* statistic = 0.006–0.65, *p* > 0.05, 6 mice, 8 odor pairs; Extended Data Fig. 7-1). A GLM for the divergence times for pre-lick tPRP found no statistically significant differences between licks and either peak or trough for γ for CA1 (*p* > 0.05, 100 observations, 97 df, *F* statistic = 0.086, *p* > 0.05, 6 mice, 8 odor pairs; Extended Data Fig. 7-1) and found a statistically significant difference between both peak and trough and licks for β CA1 (*p* < 0.05, 100 observations, 97 df, *F* statistic = 4, *p* < 0.05, 6 mice, 8 odor pairs; Extended Data Fig. 7-1). In contrast, for post-lick ztPRP, divergence for all bandwidths and for both CA1 and mPFC GLM found a statistically significant difference between both peak and trough and lick divergence (*p* < 0.001, 124 observations, 121 df, *F* statistic = 17.8–31.8, *p* < 0.001, 6 mice, 8 odor pairs; Extended Data Fig. 7-1). Asterisks denote significant differences determined *post hoc* with *t* or ranksum tests (*p* < pFDR). Extended Data Figure 7-1 provides GLM and ANOVAN statistical analysis for the data in ***C–H***.

In order to understand the relationship between licks and the power of β and γ referenced to the peak and trough of θ (tPRP) we sorted the time of occurrence of θ oscillation peaks and troughs with respect to the time of onset of adjacent licks for proficient animals. Figure 7*A*,*B* show the probability density (PD) for peak (Fig. 7*A*) and trough (Fig. 7*B*) occurrence timed with respect to adjacent licks. Consistent with studies in mPFC (Amarante et al., 2017; Amarante and Laubach, 2021) the average peaks tend to occur near the lick while the trough probability density show a bimodal distribution with peaks before and after the lick. We then calculated β and γ ztPRP time courses for peaks and troughs that occur before and after the lick (pre-lick-referenced and post-lick-referenced ztPRP). We found that the time course for pre-lick-referenced tPRP (Fig. 7*C*,*E*) tended to diverge less between rewarded and unrewarded odorants than the post-lick-referenced tPRP (Fig. 7*D*,*F*) and that the divergence was sustained for post-lick-referenced ztPRPs and transient for pre-lick-referenced ztPRPs (Fig. 7*C–F*). GLM for the average β or γ ztPRP between 0.5 and 2.5 s found statistically significant differences for both CA1 and mPFC for naive versus proficient (naive are not shown; Fig. 7), rewarded versus unrewarded odorant and pre-lick versus post-lick (*p* < 0.001, 752 observations, 736 df, *F* statistic = 91–123, *p* < 0.001, 6 mice, 8 odor pairs; Extended Data Fig. 7-1).

Additionally, we found an interesting difference between pre-lick-referenced ztPRP and post-lick-referenced ztPRP when we assessed the time for divergence between rewarded and unrewarded trials for the time courses for lick-referenced ztPRP. For post-lick-referenced ztPRP the time for divergence was smaller than the time for divergence for lick behavior for all bandwidths for both peak and trough for both CA1 and mPFC (Fig. 7*H*). In contrast, for pre-lick-referenced ztPRP in mPFC the time for divergence for both bandwidths and peak and trough did not differ from the time for divergence for lick behavior (Fig. 7*G*iii,iv). For CA1 the time for divergence for pre-lick-referenced ztPRP for both peak and trough differed from the time for divergence for lick behavior for β, but not for γ (Fig. 7*G*i,ii). A GLM for the divergence times for pre-lick tPRP found no statistically significant differences between licks and either peak or trough for all bandwidths for mPFC (*p* > 0.05, 100 observations, 97 df, *F* statistic = 0.006–0.65, *p* > 0.05, 6 mice, 8 odor pairs; Extended Data Fig. 7-1). A GLM for the divergence times for pre-lick tPRP found no statistically significant differences between licks and either peak or trough for γ for CA1 (*p* > 0.05, 100 observations, 97 df, *F* statistic = 0.086, *p* > 0.05, 6 mice, 8 odor pairs; Extended Data Fig. 7-1) and found a statistically significant difference between both peak and trough and licks for β CA1 (*p* < 0.05, 100 observations, 97 df, *F* statistic = 4, *p* < 0.05, 6 mice, 8 odor pairs; Extended Data Fig. 7-1). In contrast, for post-lick ztPRP divergence for all bandwidths and for both CA1 and mPFC GLM found a statistically significant difference between both peak and trough and lick divergence (*p* < 0.001, 124 observations, 121 df, *F* statistic = 17.8–31.8, *p* < 0.001, 6 mice, 8 odor pairs; Extended Data Fig. 7-1).

### Coordinated hippocampal-prefrontal neural activity decreased for the unrewarded odorant as the animal became proficient

Coordinated hippocampal-prefrontal neural activity supports the organization of brain rhythms and is present during a range of cognitive functions presumably underlying transfer of information between these two brain regions (Colgin, 2011; Gordon, 2011; Headley and Paré, 2017; Lisman et al., 2017). We proceeded to determine whether there were changes in coordinated neural activity between dorsal CA1 and mPFC as the animal learned to discriminate the odorants. We used two complementary methods to quantify coordinated neural activity by calculating imaginary coherence (iCoherence) and PLV, measures of coherence that are independent of volume conduction (Nolte et al., 2004; Bastos and Schoffelen, 2016; Namburi, 2021).

**Figure 8.**
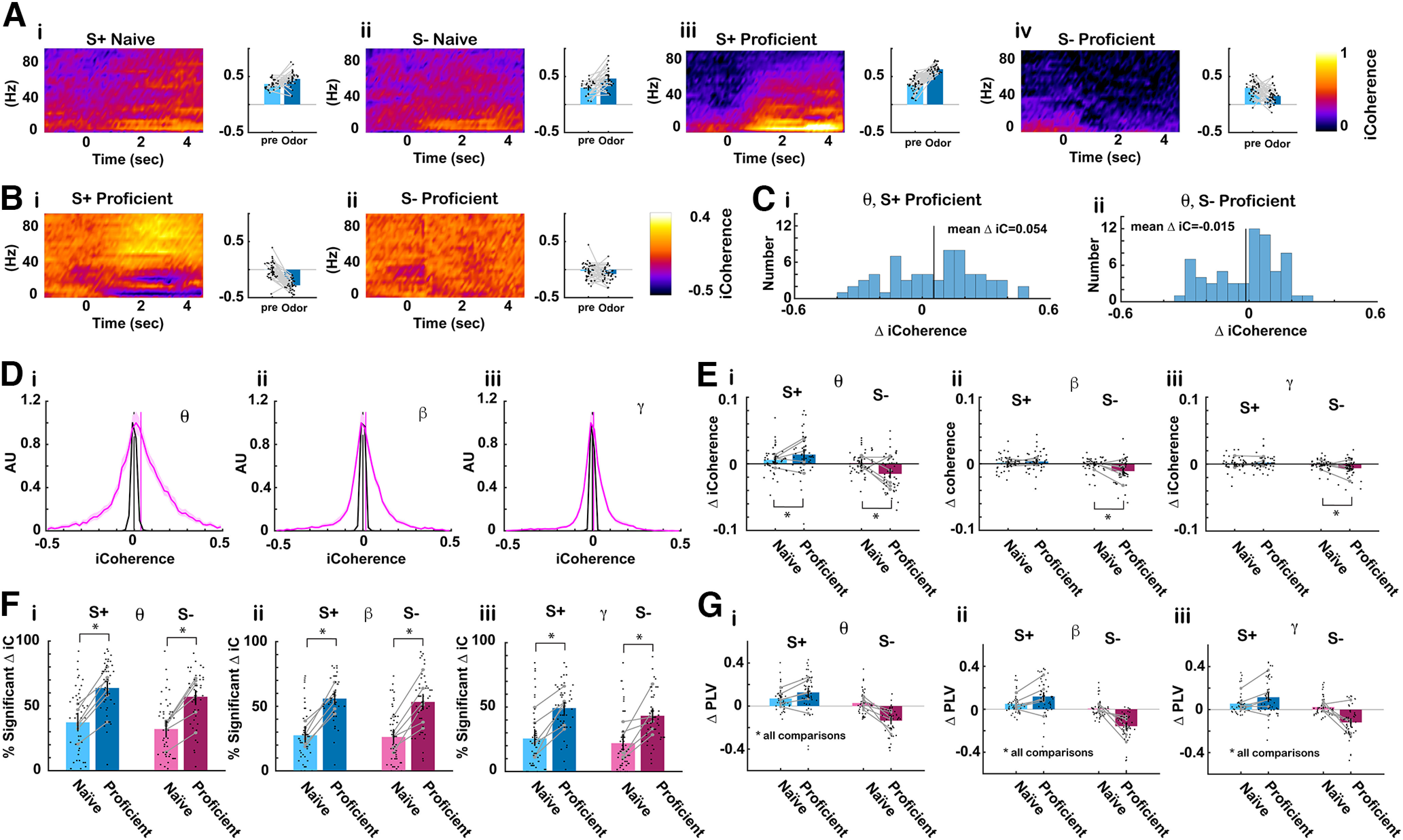
Odorant-elicited change in iCoherence and PLV decreased for the unrewarded odorant as the animal became proficient. ***A***, Example of pseudocolor plots of the average mPFC/CA1 iCoherence average trial time course for (i) S+ naive (30 trials), (ii) S– naive (27 trials), (iii) S+ proficient (33 trials), (iv) S– proficient (35 trials). Positive iCoherence means that CA1 oscillatory activity precedes the activity in mPFC because iCoherence is referenced to an electrode in the hippocampus. ***B***, iCoherence time course for the same mPFC electrode referenced to a different CA1 electrode for the same proficient trials as in ***A***. (i) S+ proficient (33 trials), (iv) S– proficient (35 trials). In this case mPFC precedes CA1. ***C***, Odorant-elicited change in θ iCoherence for all 64 electrode pairs in the same session as in ***A*** iii, iv. (i) S+ proficient (33 trials), (ii) S– proficient (35 trials). ***D***, Distribution of average iCoherence during odorant application per mouse per odorant pair. The distribution is normalized to the peak. Pink is the distribution for iCoherence during the odorant and black is the distribution for iCoherence calculated after shuffling trials. Shown is the mean bounded by the 95% CI of the distributions calculated per odorant per mouse. All conditions were included in this distribution: naive and proficient, S+ and S–. Vertical bars are the mean iCoherence values. ***E***, Summary for changes in odorant-elicited changes in iCoherence (Δ iCoherence) as the animal learns. Average Δ iCoherence is shown for the different bandwidths (per mouse per odorant pair): (ii) θ, (iii) β, (iv) γ (6 mice, 8 odor pairs, the vertical line is the 95% CI). GLM found for all bandwidths a statistically significant difference for naive versus proficient (*p* < 0.001, 188 observations, 184 df, *F* statistic = 6–12, *p* < 0.001, 6 mice, 8 odor pairs; Extended Data Fig. 8-1) and for the interaction between naive versus proficient and rewarded versus unrewarded (*p* < 0.05, 188 observations, 184 df, *F* statistic = 6–12, *p* < 0.001, 6 mice, 8 odor pairs; Extended Data Fig. 8-1). ***F***, Percent significant odorant-elicited changes in iCoherence per odorant per mouse. GLM found for all bandwidths a statistically significant difference for naive versus proficient (*p* < 0.001, 188 observations, 184 df, *F* statistic = 18.7–29.1, *p* < 0.001, 6 mice, 8 odor pairs; Extended Data Fig. 8-1). ***G***, Bar graphs showing odor-elicited change in average odorant-elicited change in phase-locking value (Δ PLV) per mouse per odor pair for (i) θ, (ii) β, (iii) γ (6 mice, 8 odor pairs, the vertical line is the 95% CI). GLM found statistically significant differences between naive versus proficient (*p* < 0.05) and for S+ versus S– and the interaction between S+ versus S– and naive versus proficient (*p* < 0.001, 192 observations, 188 df, *F* statistic = 29.5–46.5, *p* < 0.001, 6 mice, 8 odor pairs; Extended Data Fig. 8-1). Extended Data Figure 8-1 provides GLM and ANOVAN statistical analysis for the data in ***E–G***.

Figure 8*A* shows an example of a spectrogram for the time course for iCoherence for a session where a mouse was engaged in the go-no go task. The pseudocolor plots show the average iCoherence time course per trial for (Fig. 8*A*i) S+ naïve, (Fig. 8*A*ii) S– naïve, (Fig. 8*A*iii) S+ proficient, (Fig. 8*A*iv) S– proficient. For S+ proficient there is an increase in iCoherence after odorant addition compared with naive. This positive iCoherence indicates that coherent oscillations that take place in CA1 before they ensue in mPFC. Another example for a different electrode pair from the same session for the proficient animal shows that the rewarded odorant elicits a decrease in θ iCoherence to a negative value (oscillations take place earlier in mPFC; Fig. 8*B*). Figure 8*C* shows the histogram for odorant-elicited changes in θ iCoherence for all electrode pairs for the proficient mouse in this session. The distribution of the changes in iCoherence was broad and on the average the rewarded odorant elicited an increase in θ iCoherence and the unrewarded odorant elicited a smaller increase.

We proceeded to characterize iCoherence and the odorant-elicited changes in iCoherence for all sessions. Figure 8*D*, magenta bounded lines, show the distribution of average θ, β, and γ iCoherence per mouse per odor pair during the odorant administration period (0.5–2.5 s). The mean θ iCoherence illustrated by a vertical magenta line is positive, indicating that on the average θ oscillations take place earlier in CA1, consistent with findings by other investigators (Adhikari et al., 2010). Shuffling the trials results in a narrow symmetrical distribution for iCoherence centered at zero (Fig. 8*D*, black bounded lines). Figure 8*E* shows the change of iCoherence (Δ iCoherence) elicited by the rewarded (S+) and unrewarded (S–) odorants for naive and proficient mice calculated as the mean per odorant pair per mouse. Δ iCoherence decreases for the unrewarded odorant when the animal becomes proficient. A GLM analysis found for all bandwidths statistically significant differences for Δ coherence for S+ versus S– and the interaction between S+ versus S– and naive versus proficient (*p* < 0.001, 188 observations, 184 df, *F* statistic = 12.6–32.4, *p* < 0.001, 6 mice, 8 odor pairs; Extended Data Fig. 8-1).

Figure 8*G* shows the odor-elicited change in average PLV (Δ PLV) per mouse per odor pair for (Fig. 8*G*i) θ, (Fig. 8*G*ii) β, (Fig. 8*G*iii) γ. Here, we also found a negative Δ PLV for the unrewarded odorant for the proficient mice. For Δ PLV GLM found statistically significant differences for Δ PLV between naive versus proficient (*p* < 0.05), S+ versus S– and the interaction between S+ versus S– and naive versus proficient (*p* < 0.001, 192 observations, 188 df, *F* statistic = 29.5–46.5, *p* < 0.001, 6 mice, 8 odor pairs). In conclusion, both the Δ iCoherence and Δ PLV measures indicate that as the mouse becomes proficient there was a decrease in coordinated hippocampal-prefrontal neural activity for the unrewarded odorant.

### Decreased performance for homozygote and heterozygote CaMKIIα knock-out mice in the go-no go task

**Figure 9.**
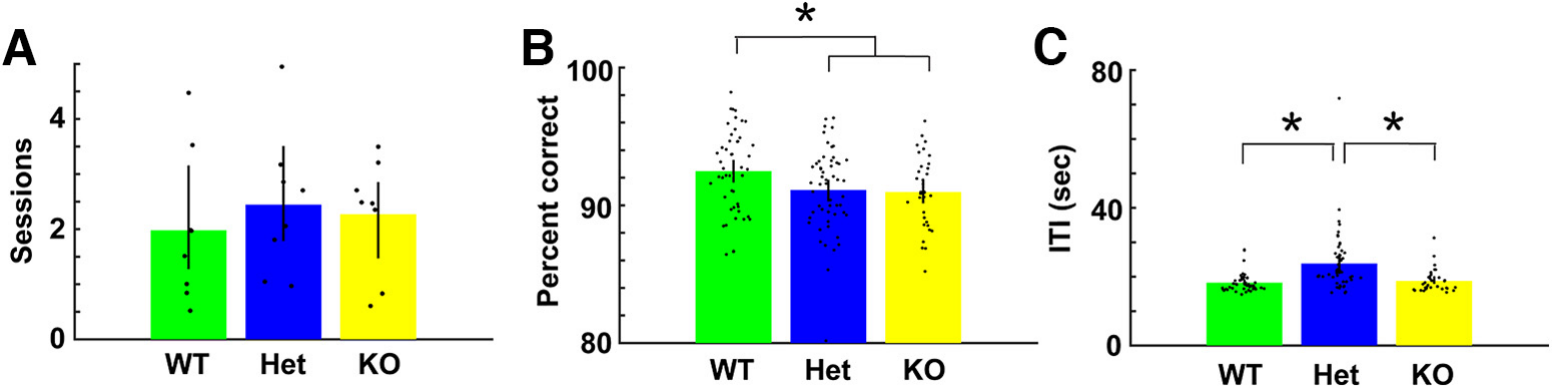
Analysis of the behavioral performance for mice of the three genotypes. ***A***, Number of sessions to criterion per odorant pair (2 blocks of 20 trials with performance >80%). There was no difference between genotypes in the number of sessions to criterion (GLM *p* > 0.05, 24 observations, 21 df, *F* statistic = 0.26, *p* > 0.05, 8 odor pairs). ***B***, Percent correct for proficient mice per odorant per mouse. GLM found a statistical difference for WT compared with both CaMKIIα Het and CaMKIIα KO (GLM *p* < 0.05, 137 observations, 134 df, *F* statistic = 3.7, *p* < 0.05, 6 mice, 8 odor pairs). ***C***, Intertrial interval (ITI) for proficient mice. GLM analysis found that the ITI for CaMKIIα Het differed from both CaMKIIα Het and CaMKIIα KO (*p* < 0.001, 137 observations, 134 df, *F* statistic = 13.5, *p* < 0.001, 6 mice, 8 odor pairs). Asterisks denote statistically significant differences evaluated with either *t* test or ranksum corrected for multiple comparisons (*p* < pFDR). Extended Data Figure 9-1 provides GLM statistical analysis for the data in ***A–C***.

Since CaMKIIα is a protein involved in LTP, we asked whether behavioral performance differed between the different CaMKIIα genotypes (WT, CaMKIIα Het and CaMKIIα KO). We trained mice from the three genotypes in the go-no go task. There was no difference in the number of sessions to criterion (Fig. 9*A*, GLM *p* > 0.05, 24 observations, 21 df, *F* statistic = 0.26, *p* > 0.05, 8 odor pairs; Extended Data Fig. 9-1). However, for proficient mice the percent correct was higher for WT compared with both CaMKIIα Het and CaMKIIα KO (Fig. 9*B*, GLM *p* < 0.05, 137 observations, 134 df, *F* statistic = 3.7, *p* < 0.05, 6 mice, 8 odor pairs; Extended Data Fig. 9-1) and the intertrial interval was larger for CaMKIIα Het (Fig. 9*B*, *p* < 0.001, 137 observations, 134 df, *F* statistic = 13.5, *p* < 0.001, 6 mice, 8 odor pairs; Extended Data Fig. 9-1).

### The strength of tPAC and peak angle variance differed between the CaMKIIα genotypes

**Figure 10.**
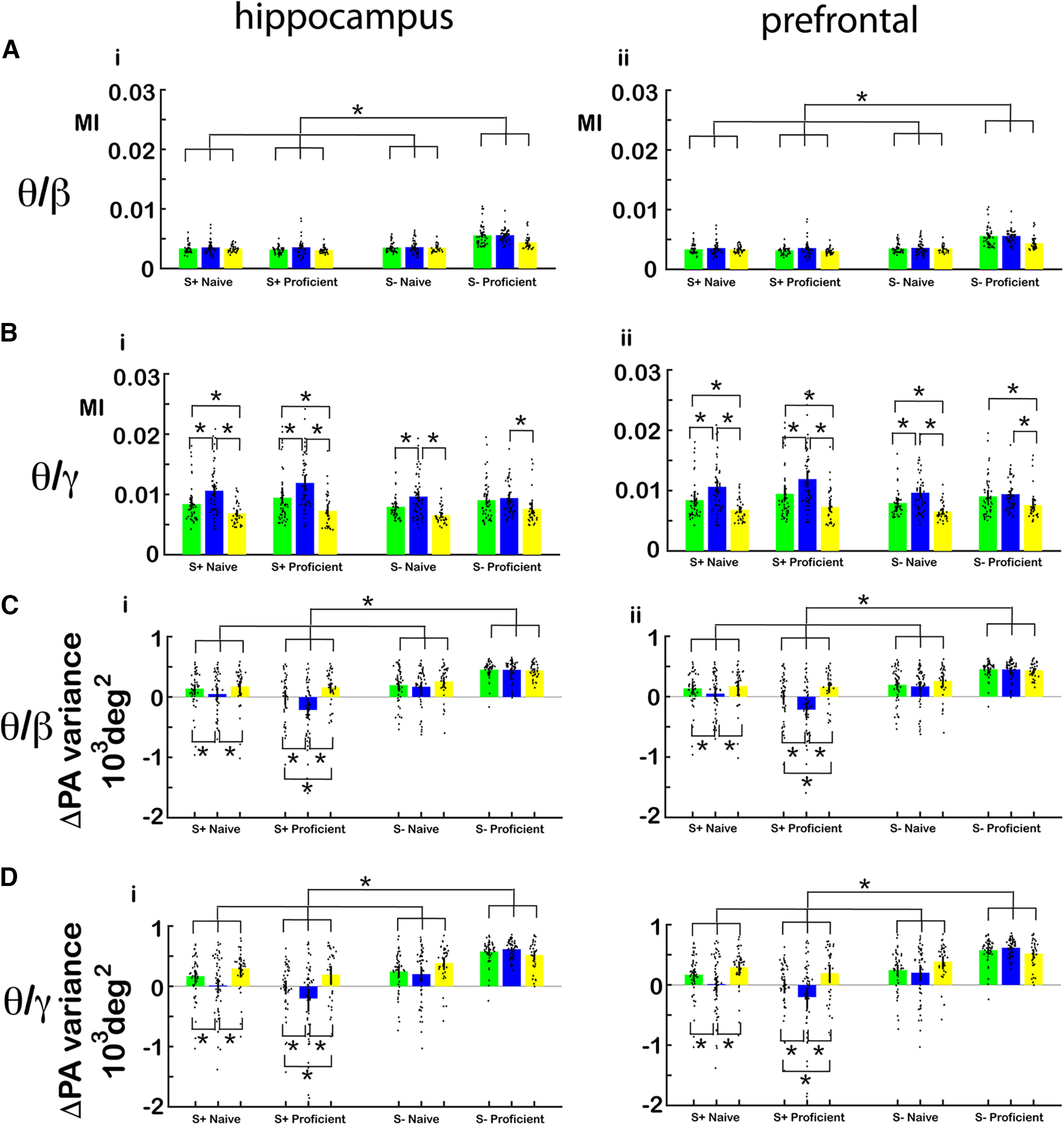
tPAC and peak angle variance per genotype. ***A***, ***B***, Bar graphs showing average MI per mouse per odor pair for each genotype, S+ and S– in naive and proficient mice for (***A***) θ/β and (***B***) θ/γ for (i) hippocampus and (ii) mPFC. The bars show the average MI, and the points are the MI per mouse per odor pair. For θ/β tPAC MI GLM found a statistically significant difference for S+ versus S–, for the interaction of WT versus KO and S+ versus S– and the interaction of WT versus KO and naive versus proficient (*p* < 0.001, 544 observations, 532 df, *F* statistic = 10.3–34.3, *p* < 0.001, 6 mice, 8 odor pairs). For θ/γ tPAC MI GLM found a statistically significant difference between WT versus KO and WT versus Het (*p* < 0.01) and for the interaction of WT versus Het and S+ versus S– (*p* < 0.05, 544 observations, 532 df, *F* statistic = 10.3–34.3, *p* < 0.001, 6 mice, 8 odor pairs; Extended Data Fig. 10-1). ***C***, ***E***, Bar graphs showing average peak angle variance per mouse per odor pair for each genotype, S+ and S– in naive and proficient mice for (***A***) θ/β and (***B***) θ/γ for (i) hippocampus and (ii) mPFC. For β tPAC MI GLM found a statistically significant difference for WT versus Het (*p* < 0.001) and WT versus KO (*p* < 0.05), S+ versus S–, (*p* < 0.001) and naive versus proficient (*p* < 0.05, 544 observations, 532 df, *F* statistic = 20.6, *p* < 0.001, 6 mice, 8 odor pairs; Extended Data Fig. 10-1). For γ tPAC MI GLM found a statistically significant difference for WT versus Het (*p* < 0.05) and WT versus KO (*p* < 0.05), S+ versus S–, (*p* < 0.001) and naive versus proficient (*p* < 0.05, 544 observations, 532 df, *F* statistic = 18.8, *p* < 0.001, 6 mice, 8 odor pairs; Extended Data Fig. 10-1). Extended Data Figure 10-1 provides GLM statistical analysis for the data in ***A–D***.

We then asked whether tPAC in the hippocampus and mPFC differs between the different genotypes. When we compared strength of tPAC, measured by the MI, and the peak angle variance between genotypes (WT, CaMKIIα Het and CaMKIIα KO), we found significant differences. Figure 10*A*,*B* shows the average MI per mouse per odorant pair for β (Fig. 10*A*i), high γ (Fig. 10*B*i) for hippocampus and for β (Fig. 10*A*ii) and γ (Fig. 10*B*ii) for PFC. For β tPAC GLM found a statistically significant difference for MI for the interaction between WT versus CaMKIIα KO and S+ versus S– in both the hippocampus and mPFC (*p* < 0.001, 544 observations, 532 df, *F* statistic = 10.3–34.3, *p* < 0.001, 6 mice, 8 odor pairs; Extended Data Fig. 10-1). For high γ tPAC there was an increase in MI for CaMKIIα Het and a decrease in MI for CaMKIIα KO compared with WT and GLM analysis found a statistically significant difference between WT versus CaMKIIα KO and WT versus CaMKIIα Het for both hippocampus and mPFC (*p* < 0.001, 544 observations, 532 df, *F* statistic = 10.3–34.3, *p* < 0.001, 6 mice, 8 odor pairs; Extended Data Fig. 10-1).

Figure 1*C*,*D* shows average peak angle variance per mouse per odorant pair for β (Fig. 1*C*i), high γ (Fig. 1*E*i) in the hippocampus and (Fig. 1*C*ii) β, (Fig. 1*E*ii) high γ in PFC. For proficient mice, the peak angle variance for the rewarded odorant decreased for CaMKα Het and increased for CaMKα KO. GLM analysis found a statistically significant difference for WT versus CaMKIIα Het, WT versus CaMKIIα KO, naive versus proficient and S+ versus S– for θ/β and θ/γ for both the hippocampus and mPFC (*p* < 0.05, 544 observations, 532 df, *F* statistic = 20.6, *p* < 0.001, 6 mice, 8 odor pairs; Extended Data Fig. 10-1).

### The accuracy for decoding the contextual identity of the odorant decreased in the CaMKIIα knock-out mouse and was correlated with percent correct discrimination

**Figure 11.**
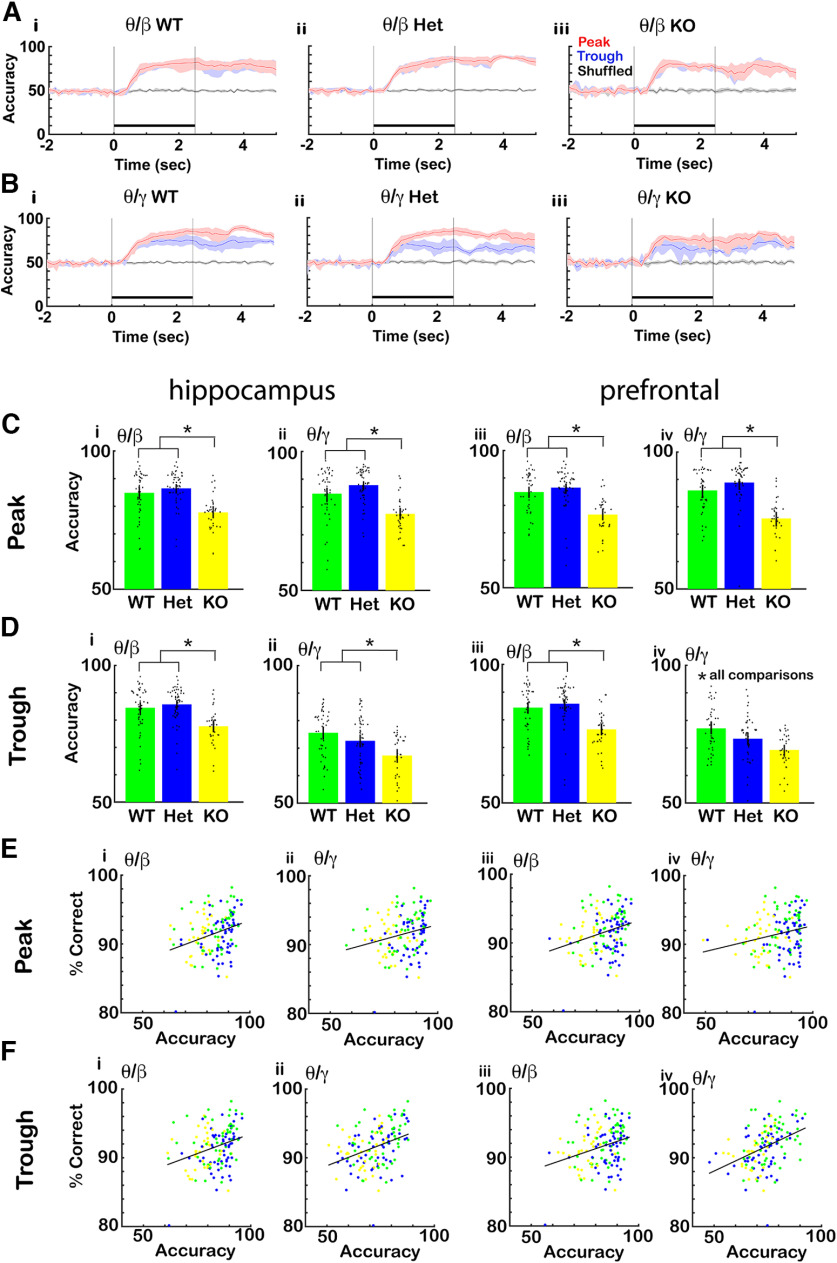
The accuracy for decoding the contextual identity of the odorant from tPRP decreased in the CaMKIIα knock-out mouse and was correlated with percent correct discrimination. ***A***, ***B***, Examples for one mouse of the time course for the accuracy of odorant identification by LDA trained using CA1 tPRP for the EAPA odor pair. ***A***, β tPRP. ***B***, γ tPRP. (i) WT. (ii) Het. (iii) KO. Shadow: confidence interval, black bar: odorant application. ***C***, ***D***, Bar graphs showing the differences in discriminant accuracy between the different genotypes. ***C***, Accuracy for peak tPRP for (i) θ/β in the hippocampus, (ii) θ/γ in the hippocampus, (iii) θ/β in mPFC, (iv) θ/γ in mPFC. ***D***, Accuracy for through for (i) θ/β in the hippocampus, (ii) θ/γ in the hippocampus, (iii) θ/β in mPFC, (iv) θ/γ mPFC. The bars show the average accuracy, and the points are the accuracy per mouse per odor pair. The vertical bars show the confidence interval. For β tPRP LDA GLM found statistically significant differences between WT and KO for all conditions (*p* < 0.001) and between WT and Het for γ trough tPRP (*p* < 0.05, 756 observations, 744 df, *F* statistic = 9.9–34.3, *p* < 0.001, 6 mice, 8 odor pairs; Extended Data Fig. 11-1). Asterisks show significant *p* values (*p*<pFDR) for *post hoc* pairwise tests. ***E***, Relationship for proficient mice between percent correct in the go-no go behavior and accuracy of odor identification by the LDA decoding algorithm shown per mouse per odor pair (6 mice, 8 odor pairs). The correlation coefficients were: ***E*** (i) 0.3, ***E*** (ii) 0.24, ***E*** (iii) 0.29, ***E*** (iv) 0.23, ***F*** (i) 0.3, ***F*** (ii) 0.36, ***F*** (iii) 0.29, ***F*** (iv) 0.40, and the *p* value for significance was *p* < 0.01. Lines are best fit lines. Extended Data Figure 11-1 provides GLM statistical analysis for the data in ***C–F***.

The differences in tPAC between CaMKIIα genotypes raises the question whether decoding of contextual identity from tPRP is altered in the CaMKIIα KO and the CaMKIIα Het mice. Figure 11 shows the results of our comparison of decoding accuracy between genotypes. Figure 11*A*,*B* shows examples for proficient mice of the time course for the accuracy of decoding of odorant contextual identity by LDA trained using tPRP for the EAPA odor pair [Fig. 11*A*, θ/β tPRP; Fig. 11*B*, θ/γ tPRP, (i) WT, (ii) Het, (iii) KO]. For the WT and CaMKIIα Het mice the accuracy increases monotonously during the odorant epoch (Fig. 11*A*i,ii, *B*i,ii) while for the CaMKIIα KO mouse the accuracy reaches a maximum value after 1 s and then decreases slightly for the rest of the odorant epoch (Fig. 11*A*iii, *B*iii). Figure 11*C*,*D* shows the differences in decoding accuracy between the different genotypes assessed in the window from 1.5 to 2.5 s after addition of the odorant. For both β and γ peak or trough tPAC decoding for both the hippocampus and the mPFC decoding accuracy was lowest for CaMKIIα KO. In addition, for γ trough tPRP decoding for mPFC the accuracy was highest for WT, and decreased for both CaMKIIα Het and CaMKIIα KO mice (Fig. 11*D*iv). For β tPRP LDA GLM found statistically significant differences for decoding accuracy between WT and KO for all conditions (*p* < 0.001) and between WT and Het for peak γ tPRP in the hippocampus, trough γ tPRP in mPFC (*p* < 0.05, 137 observations, 134 df, *F* statistic = 9.9–34.3, *p* < 0.001, 6 mice, 8 odor pairs; Extended Data Fig. 11-1).

**Figure 12.**
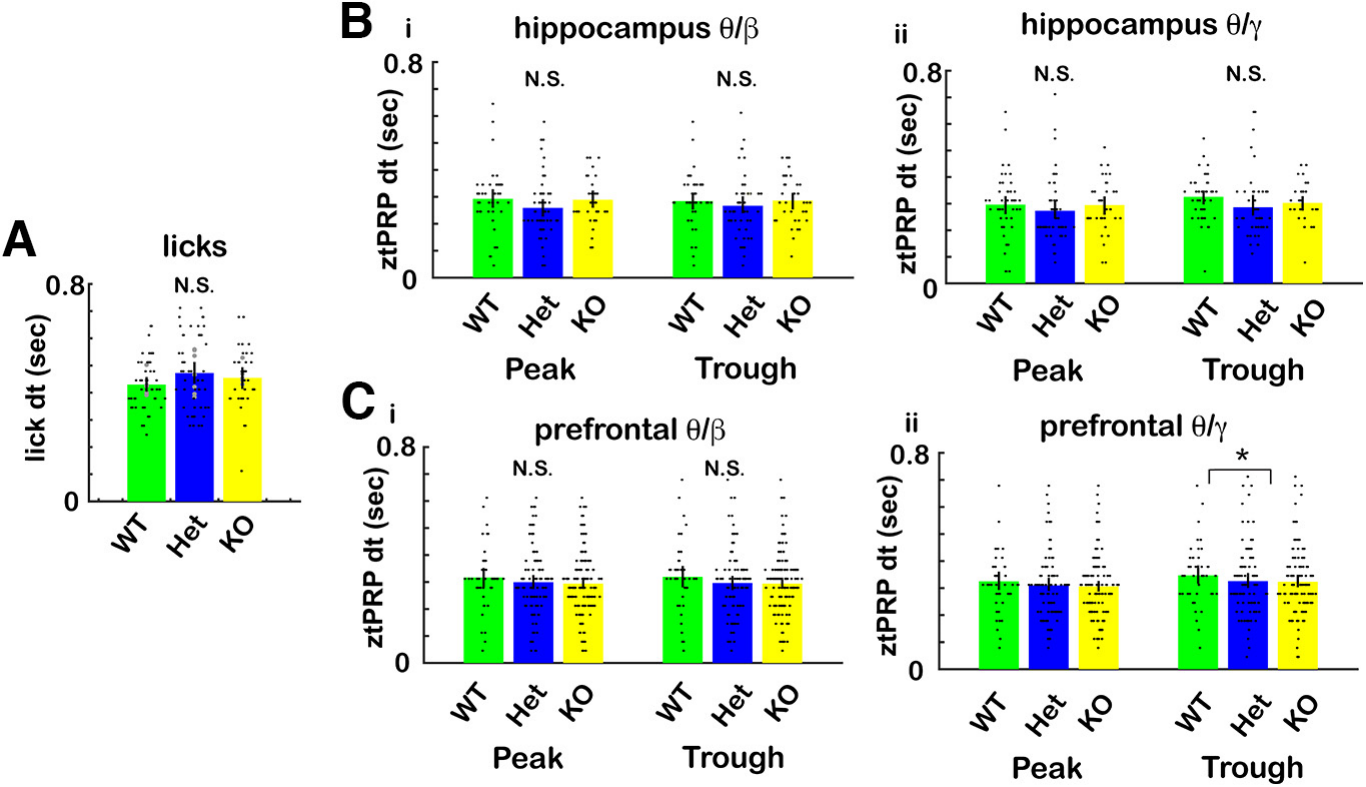
Divergence times between the ztPRP time courses for rewarded and unrewarded trials for the different CaMKIIα genotypes. Divergence times between rewarded and unrewarded ztPRP and licks were calculated from the ranksum *p* value time courses as shown in Figure 6 (also see Materials and Methods). ***A***, Divergence times for licks. A GLM for the divergence times did not find statistically significant differences between genotypes for licks (*p* > 0.05, 127 observations, 124 df, *F* statistic = 1.5, *p* > 0.05, 6 mice, 8 odor pairs; Extended Data Fig. 12-1). ***B***, ***C***, Divergence times for ztPRP. ***B***, CA1. ***C***, mPFC. (i) β tPRP. (ii) γ tPRP. A GLM for the divergence times did not find statistically significant differences between genotypes for β or γ ztPRP (*p* > 0.05, 252 observations, 246 df, *F* statistic = 0.5–1, *p* > 0.05, 6 mice, 8 odor pairs; Extended Data Fig. 12-1). Extended Data Figure 12-1 provides GLM and ANOVAN statistical analysis for the data in ***A–C***.

Finally, we did not find differences between CaMKIIα genotypes for divergence times between rewarded and unrewarded ztPRP and or lick time courses (Fig. 12, divergence times were calculated as in Fig. 6). GLM for the divergence times did not find statistically significant differences between genotypes for licks (*p* > 0.05, 127 observations, 124 df, *F* statistic = 1.5, *p* > 0.05, 6 mice, 8 odor pairs; Extended Data Fig. 12-1). A GLM for the divergence times did not find statistically significant differences between genotypes for β or γ ztPRP (*p* > 0.05, 252 observations, 246 df, *F* statistic = 0.5–1, *p* > 0.05, 6 mice, 8 odor pairs; Extended Data Fig. 12-1).

### Relationship between tPRP decoding and behavior and decision-making times across genotypes

We then asked whether there was a relationship between contextual odorant identity decoding accuracy and percent correct performance for proficient mice in the go-no go task. Figure 11*E*,*F* shows that there were statistically significant correlations between decoding accuracy and percent correct performance for all the different conditions. The correlation coefficients were as follows: 0.3 for hippocampal peak β tPRP, 0.24 for hippocampal peak γ tPRP, 0.29 for mPFC peak β tPRP, 0.23 for mPFC peak γ tPRP, 0.3 for hippocampal trough β tPRP, 0.36 for hippocampal trough γ tPRP, 0.29 for mPFC trough β tPRP and 0.40 for mPFC trough γ tPRP, and the *p* value for significance of the correlation coefficient was *p* < 0.01. This indicates that the decoding accuracy obtained with tPRP is related to behavioral performance.

### Coherent hippocampal-prefrontal neural activity differed between CaMKIIα genotypes

**Figure 13.**
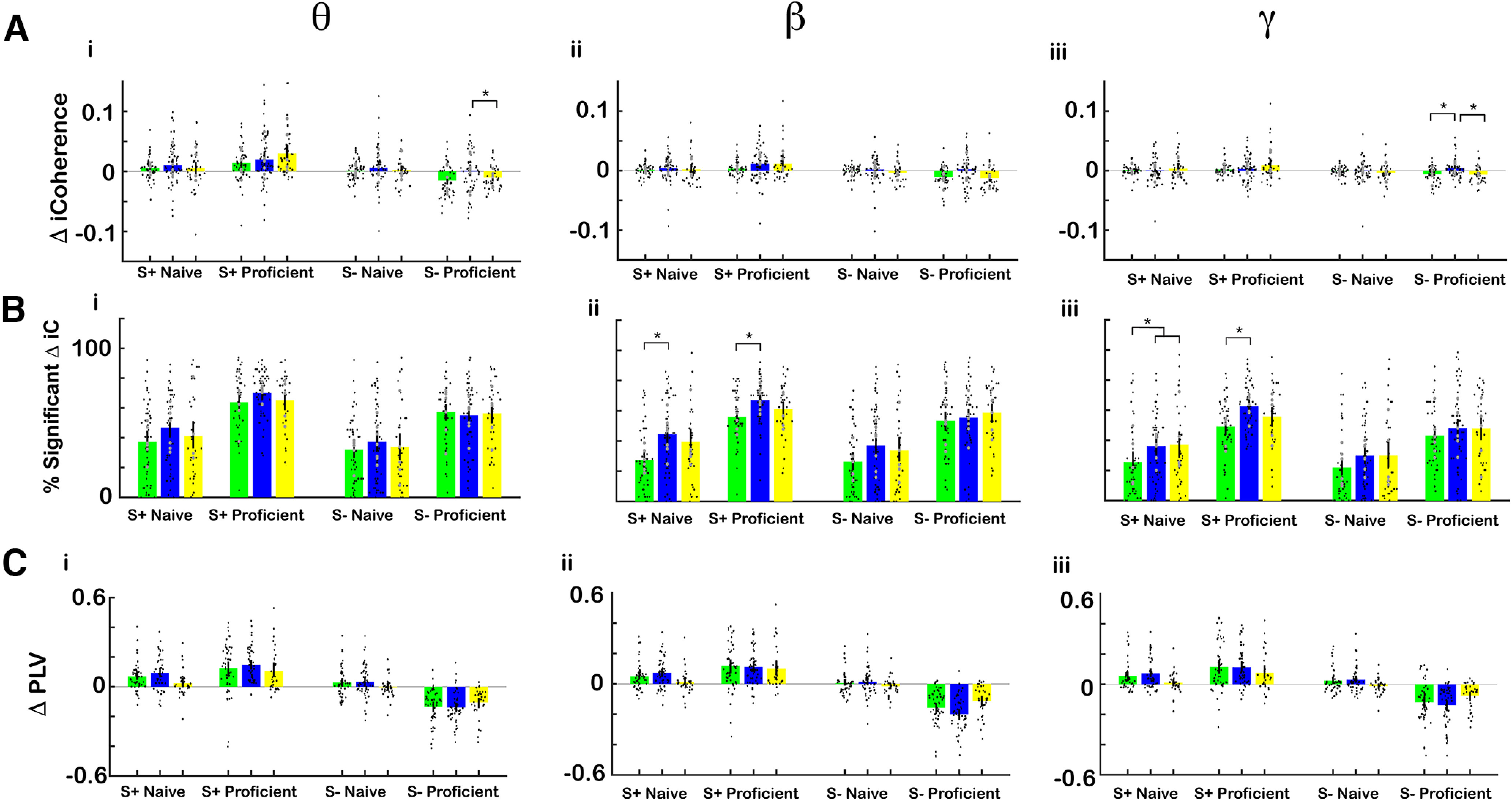
There were small differences in odorant-elicited change in iCoherence between CaMKIIα genotypes. ***A***, Summary bar graphs comparing the odorant-elicited change in iCoherence (Δ iCoherence) for the different genotypes. (i) θ, (ii) β, (iii) γ. The bars show the average Δ iCoherence, and the points are Δ iCoherence per mouse per odor pair. The vertical bars show the confidence interval. For θ Δ iCoherence GLM found a statistically significant difference between WT and KO (*p* < 0.05, 544 observations, 532 df, *F* statistic = 5.35, *p* < 0.001, 6 WT mice, 7 Het mice and 5 KO mice, 8 odor pairs; Extended Data Fig. 13-1). For γ Δ iCoherence, GLM found a statistically significant difference for the interaction between WT and KO and rewarded versus unrewarded odorant (*p* < 0.05, 544 observations, 532 df, *F* statistic = 2.76, *p* < 0.05, 6 WT mice, 7 Het mice and 5 KO mice, 8 odor pairs; Extended Data Fig. 13-1). Asterisks show significant *p* values (*p*<pFDR) for *post hoc* pairwise tests. ***B***, Percent significant odorant-elicited changes in iCoherence per odorant per mouse. For β Δ iCoherence GLM found a statistically significant difference between WT and Het (*p* < 0.05, 544 observations, 532 df, *F* statistic = 18.1, *p* < 0.001, 6 WT mice, 7 Het mice and 5 KO mice, 8 odor pairs; Extended Data Fig. 13-1). For γ Δ iCoherence, GLM found a statistically significant difference for the interaction between WT and het and rewarded versus unrewarded odorant (*p* < 0.05, 544 observations, 532 df, *F* statistic = 14.7, *p* < 0.001, 6 WT mice, 7 Het mice and 5 KO mice, 8 odor pairs; Extended Data Fig. 13-1). ***C***, Summary bar graphs comparing change in Δ PLV for the different genotypes. (i) β, (ii) θ, (iii) γ. For Δ PLV GLM found no statistically significant differences between WT and KO, and for the interaction of WT versus KO and S+ versus S– (*p* > 0.05, 512 observations, 500 df, *F* statistic = 25.5–39.9, *p* < 0.001, 6 mice, 8 odor pairs; Extended Data Fig. 13-1). Extended Data Figure 13-1 provides GLM and ANOVAN statistical analysis for the data in ***A–C***.

We asked whether there were differences for coordinated hippocampal-prefrontal neural activity for the different CaMKIIα genotypes. Figure 13*A* shows the odorant-elicited changes in Δ iCoherence for the different genotypes. For θ Δ iCoherence GLM found a statistically significant difference between WT and KO (*p* < 0.05, 544 observations, 532 df, *F* statistic = 5.35, *p* < 0.001, 6 WT mice, 7 Het mice and 5 KO mice, 8 odor pairs; Extended Data Fig. 13-1). However, these were relatively small differences of θ Δ iCoherence between genotypes and *post hoc* tests did not yield significant differences between CaMKIIα KO and WT. Furthermore, the percent of electrode pairs that showed a significant Δ iCoherence was higher for Hets compared with WT for the rewarded odorant for β and γ Δ iCoherence (Fig. 13*B*ii,iii). For β Δ iCoherence GLM found a statistically significant difference between WT and Het (*p* < 0.05, 544 observations, 532 df, *F* statistic = 18.1, *p* < 0.001, 6 WT mice, 7 Het mice and 5 KO mice, 8 odor pairs; Extended Data Fig. 13-1). For γ Δ iCoherence, GLM found a statistically significant difference for the interaction between WT and Het and rewarded versus unrewarded odorant (*p* < 0.05, 544 observations, 532 df, *F* statistic = 14.7, *p* < 0.001, 6 WT mice, 7 Het mice and 5 KO mice, 8 odor pairs; Extended Data Fig. 13-1).

Δ PLV did not show any differences between genotypes (Fig. 13*C*). The GLM found no statistically significant differences for Δ PLV between WT and KO or WT and Het (*p* > 0.05, 512 observations, 500 df, *F* statistic = 25.5–39.9, *p* < 0.001, 6 mice, 8 odor pairs).

## Discussion

Santiago Ramon y Cajal described the hippocampus as a quaternary region of the olfactory system (Cajal, 1904), but subsequent studies showed that the hippocampus is involved in learning and memory in nonolfactory tasks (Nakazawa et al., 2004). However, the hippocampus does play a role in olfactory learning. Indeed, calbindin-expressing pyramidal cells in dorsal CA1 develop selective responses to odorants as the animal becomes proficient in the go-no go olfactory discrimination task (Y Li et al., 2017). Furthermore, studies of oscillatory neural activity implicate directional coupling from the OB to the hippocampus in go-no go learning (Martin et al., 2007; Gourévitch et al., 2010). In addition, Granger directionality analysis found that distinct low-frequency oscillation bandwidths link the OB and hippocampus (Nguyen Chi et al., 2016). Interestingly, mPFC has also been proposed as a downstream brain area coupled with the OB. Coupling of low-frequency oscillations between the OB and mPFC increases during freezing periods in auditory conditioned fear learning (Moberly et al., 2018); mPFC neurons represent odor value (Wang et al., 2020) and at rest there is strong coupling of low-frequency oscillations for OB-hippocampus and OB-mPFC (Mofleh and Kocsis, 2021). Furthermore, β and θ synchrony between mPFC and olfactory regions was elevated as rats switched their attention to odors (Cansler et al., 2022). Taken together with the fact that Losacco and colleagues showed that when mice become proficient in discriminating odorants in the go-no go task contextual odorant identity can be decoded from β and γ tPRP (Losacco et al., 2020), these findings raise the question whether coupled oscillations in mPFC and the hippocampus play a role in olfactory discrimination in go-no go associative learning.

In this study, we found that as animals became proficient in the go-no go task there was an increase in the variance of the peak angle for β and high γ tPAC for the unrewarded odorant (Fig. 2) accompanied by a decrease in tPRP for this odorant (Figs. 3, 6) in CA1 and mPFC. This decrease in tPRP for S– was accompanied by a small increase in tPRP for S+ resulting in a sharp increase in accuracy for decoding of the contextual odorant identity for the proficient mouse (Fig. 5). Furthermore, divergence in tPRP between rewarded and unrewarded trials took place before divergence in lick behavior (Figs. 6, 7). When we tested CaMKIIα KO mice we found a decrease in the accuracy of decoding of contextual odorant identity (Fig. 11). Finally, the behavioral performance for CaMKIIα KO and CaMKIIα Het mice was lower than the performance of WT mice (Fig. 9) and the accuracy for decoding of contextual odorant identity from tPAC correlated with behavioral performance (Fig. 11). Odor-elicited changes in iCoherence decreased for the unrewarded odorant as the animal became proficient (Fig. 8). These findings are consistent with a role for coordinated oscillatory neuronal activity in the hippocampal-mPFC axis in the go-no go olfactory discrimination task.

θ Frequency stimulation, eliciting intrinsic γ frequency oscillations, is known to be essential for LTP (Bliss and Lomo, 1973; Larson and Munkácsy, 2015; Butler et al., 2016) and tPAC of higher frequency bursts is thought to be important for information transfer between brain regions which is thought to be essential for learning (Fries, 2005). In the hippocampus, high-frequency oscillations at different phases of θ carry different information. Indeed, Siegle and Wilson (2014) showed that mice increased performance in the encoding epoch for a spatial navigation task when parvalbumin interneurons were stimulated at the peak of θ whereas when these interneurons were stimulated at the trough of θ mice increased performance in the retrieval epoch. Interestingly, here we find that as the animal becomes proficient the variance of the peak angle of tPAC increases substantially for the unrewarded odorant (Fig. 2). As a result, if the γ or β frequencies were being read by a downstream observer at a fixed angle, the information conveyed by oscillations elicited by the rewarded odorant would be missed because of the constantly changing θ phase angle. In contrast, the peak angle variance for the rewarded odorant is small, and presumably this would result in more faithful reading of this information. This is likely what underlies the increased accuracy for decoding the contextual odorant identity from β and γ tPRP in the proficient mouse (Fig. 5). Importantly, respiration-coupled oscillations aid the exchange of information between OB and the hippocampus (Nguyen Chi et al., 2016). The high-frequency (6–14 Hz) CA1 θ oscillations studied here may be caused by θ oscillatory input from the OB entrained by high-frequency sniffing of the proficient animal in the go-no go task (A Li et al., 2015), or may be because of complex interactions of the olfacto-hippocampal circuit. Future studies are needed to determine the precise role of sniffing versus olfacto-hippocampal interactions in setting θ oscillations in CA1 during the go-no go task.

Full genetic knock-out of CaMKIIα results in impairments in hippocampus-dependent cognitive tasks and impaired LTP and LTD in adult mice (Silva et al., 1992b; Coultrap et al., 2014). In contrast, although CaMKIIα Het mice share some behavioral deficits with the full CaMKIIα KO including deficient learning and memory in the Morris water maze (Silva et al., 1996; Frankland et al., 2001), adult CaMKIIα Het mice have additional working memory deficits such as repeat entry errors in the radial arms version of the Morris water maze (Yamasaki et al., 2008; Matsuo et al., 2009). In addition, while young (postnatal day 12–16) CaMKIIα Het show LTP of the same magnitude as WT mice in CA1, young adult mice display impaired basal synaptic transmission, but do not have a deficit in LTP (Goodell et al., 2016) suggesting the development of compensatory mechanisms for LTP in the Het. tPAC plays a role in hippocampal learning and memory (Siegle and Wilson, 2014) raising the question whether the CaMKIIα Het and CaMKIIα KO have deficits in cross frequency coupling.

Here, we performed to our knowledge the first study to determine whether CaMKIIα-deficient mice display altered tPAC. We find that the accuracy for decoding of contextual odorant identity from β and γ tPRP is decreased in CaMKIIα KO mice, but not in CaMKIIα Hets (with the exception of a decrease accuracy for mPFC γ trough tPRP decoding; Fig. 11). Interestingly, the strength of tPAC, measured as the MI, was highest for γ tPAC CaMKIIα Hets compared with both WT and CaMKIIα KO (Fig. 10*B*). For CaMKIIα Hets the peak angle variance decreased significantly for the rewarded odorant when the animal became proficient (Fig. 1*C*,*D*). If a downstream neural observer is evaluating contextual odorant identity by observing β or γ frequency bursts in phase with θ oscillations these changes in tPAC for CaMKIIα Hets would tend to increase the ability to discriminate (albeit with smaller accuracy for high γ trough). Thus, the differences found for tPAC for CaMKIIα Hets may be compensatory leading to no difference in decoding accuracy between WT and CaMKIIα Hets (Fig. 11). This would agree with the interpretation by Goodell et al., who indicated that LTP deficits in CaMKIIα Hets are restored by compensatory changes during development (Goodell et al., 2016). Finally, given that CaMKIIα Hets have a phenotype reminiscent of schizophrenia it is interesting that resting-state γ tPAC has been found to be increased in patients with schizophrenia (Won et al., 2018). These authors speculate that increased tPAC may be related to the compensatory hyperarousal patterns of the dysfunctional default-mode network in schizophrenia.

Whether the tPAC/tPRP changes found in CaMKIIα Het and CaMKIIα KO mice are because of the decreased expression of CaMKIIα protein or to developmental changes in circuits known to change in these mice such as in the dentate gyrus is an open question that will require future studies with temporally and spatially restricted changes in CaMKIIα activity. The global CaMKIIα will alter plasticity of pyramidal neurons in brain regions other than CA1. Furthermore, a decrease in CaMKIIα expression may alter the postsynaptic regulation by CaMKIIα of inhibitory synapse transmission onto the pyramidal neurons (Udakis et al., 2020; Cook et al., 2021). Finally, a subpopulation of granule cells that play a role in odorant discrimination in the go/no go task and are involved in generating the γ frequency oscillatory activity in the bulb express CaMKIIα (Malvaut et al., 2017). The OB granule cells may be involved in changing coordinated oscillations between the OB and hippocampus in the CaMKIIα KO/Het mice.

Finally, there was a robust decrease of ∼30% in the accuracy for decoding contextual odorant identity from tPRP for the CaMKIIα KO mouse (Fig. 11*D*), but the decrease in behavioral performance in CaMKIIα KO was small (∼1.5%; Fig. 9*B*). The small change in behavioral performance raises the question whether CaMKIIα plays a minor role in learning in the go-no go task. It is possible that CaMKIIα is indeed not involved in olfactory go-no go learning. On the other hand, studies of the role of CaMKIIα in learning and memory indicate that in the CaMKIIα KO there may be compensatory effects from the activation of other isoforms (Zalcman et al., 2018) as well as compensatory developmental changes in molecular mechanisms or circuits involved in learning in the CaMKIIα KO.

In conclusion, we found that as the mouse learns to differentiate odorants in the go-no go associative learning task there are changes in tPAC that result in an increase of the accuracy of decoding of the contextual odorant identity from tPRP. Finally, the accuracy of decoding the contextual odorant identity from tPRP decreased in the CaMKIIα KO, but did not decrease in the CaMKIIα Het, and this decoding accuracy correlated with behavioral performance across genotypes.

10.1523/ENEURO.0259-22.2022.f2-1 Extended Data Figure 2-1 GLM and ANOVAN for panels D to E in Figure 2. Download Figure 2-1, TXT file.

10.1523/ENEURO.0259-22.2022.f3-1 Extended Data Figure 3-1 GLM and ANOVAN for panels D to E in Figure 3. Download Figure 3-1, TXT file.

10.1523/ENEURO.0259-22.2022.f4-1 Extended Data Figure 4-1 GLM and ANOVAN for panels B and C in Figure 4. Download Figure 4-1, TXT file.

10.1523/ENEURO.0259-22.2022.f5-1 Extended Data Figure 5-1 GLM and ANOVAN for panels B and C in Figure 5. Download Figure 5-1, TXT file.

10.1523/ENEURO.0259-22.2022.f6-1 Extended Data Figure 6-1 GLM and ANOVAN for panels D-G in Figure 6. Download Figure 6-1, TXT file.

10.1523/ENEURO.0259-22.2022.f7-1 Extended Data Figure 7-1 GLM and ANOVAN statistical analysis for panels C-H in Figure 7. Download Figure 7-1, TXT file.

10.1523/ENEURO.0259-22.2022.f8-1 Extended Data Figure 8-1 GLM and ANOVAN statistical analysis for panels E-G in Figure 8. Download Figure 8-1, TXT file.

10.1523/ENEURO.0259-22.2022.f9-1 Extended Data Figure 9-1 GLM statistical analysis for the data in Figure 9. Download Figure 9-1, TXT file.

10.1523/ENEURO.0259-22.2022.f10-1 Extended Data Figure 10-1 GLM statistical analysis for Figure 10. Download Figure 10-1, TXT file.

10.1523/ENEURO.0259-22.2022.f11-1 Extended Data Figure 11-1 GLM statistical analysis for panels C-F in Figure 11. Download Figure 11-1, TXT file.

10.1523/ENEURO.0259-22.2022.f12-1 Extended Data Figure 12-1 GLM and ANOVAN statistical analysis for the data in panels A-C in Figure 12. Download Figure 12-1, TXT file.

10.1523/ENEURO.0259-22.2022.f13-1 Extended Data Figure 13-1 GLM and ANOVAN statistical analysis for panels A-C in Figure 13. Download Figure 13-1, TXT file.

## Synthesis

Reviewing Editor: Christine Portfors, Washington State University Decisions are customarily a result of the Reviewing Editor and the peer reviewers coming together and discussing their recommendations until a consensus is reached. When revisions are invited, a fact-based synthesis statement explaining their decision and outlining what is needed to prepare a revision will be listed below. The following reviewer(s) agreed to reveal their identity: NONE.

This is a strong manuscript and an important advance by establishing relationships between frontal cortex and the hippocampus during olfactory learning with the end goal of understanding if their coupled oscillations may contribute to successful odor learning and discrimination performance. Studies looking into frontal cortex activity in context of olfaction are rare and on that feature alone this manuscript can be seen as an advance. yet here the authors also establish links between the frontal cortex and hippocampus and how their within and between structure dynamics and coupling may lend themselves to successful odor discrimination learning. However, as noted below the authors should elaborate on the KO mice performance and examine the perspective that just because there is a statistically significant difference, this doesn't necessarily mean a functional difference.

Specific comments:

I found the first half of the paper in WT mice to be strong and of fundamental importance / significance. I do though have a question I'd appreciate the authors to dive into with regards to the latter half of the manuscript which focuses on the possible changes in network dynamics and how this may relate to behavior in the CamKiia KO mice. From my view, the KO and Het mice are very strong performers behaviorally - both in terms of learning the task and also in their discrimination performance. WT and KO mice differed, on average, by <2% in their % of correct responding and no mice performed <85% correct. A <2% reduction in performance in this task would translate into ∼1 additional error out of 10 blocks of performance in a session. And while the statistics do indicate significance, I think stepping back from this result I would interpret these data more-so from the glass is half full lens - that behavioral performance is quite intact and that you do not need CamKiia to engage in quite impressive olfactory discriminations. Yet the data are interpreted (correctly, based on the statistical outcome) that there is a reduction in discrimination performance and then later in the paper this used as a way to correlate the many interesting changes in some of the LFP metrics observed in the KO and Het mice. As the authors discuss, there are many effects of globally reduced CamKiia expression that other groups have published, on both behavior and physiology. So, given that in these mice there are alterations in several mPFC and CA1 LFP metrics, yet despite this behavior is impressively strong relative to what would see in a highly perturbed state, what does this mean about the functional significance of the LFP metrics reported herein?

Minor:

Line 317, when first introducing MI in the results, the authors may want to adjust “measure of how localized high frequency firing is within the phase of theta” to instead say “measure of how localized high frequency OSCILLATION is within phase of theta” since of course here the comparison to the carrier signal is an oscillation, not spikes.

Could the authors comment upon how temporally aligned the lick signal is relative to the physiology signals in their set-up? From the methods it seems the licking in this set-up is acquired by a different DAQ from the Intan system used for the LFPs and this may impose some forward or lag (even if just a few ms) into the lick signal relative to the LFPs. Or was the lick contact sensor 0ms lag when relayed into the Intan board? How was possible lag in the matching of these signals accounted for when analyzing licking-triggered/related LFPs?

Table 2 would benefit from a footnote to help readers know what the total number of mice from each genotype was which contributed data to the study (of the 18 in total). A quick glance at the table may give the impression otherwise that there were many mice used.

Might the authors be able to comment in the methods regarding approximately what layer(s) the tetrode tips were localized? Deep or superficial IL for instance?

Just a note that Cansler 2021 is published now, PMID: 35511680

There is a “key resources” table in the Materials and Methods. It could be 'new' Table 1, but as is does not have any formal nomenclature linking it in the manuscript.

## Author Response

We would like to thank the reviewers. Below we include a point-by-point reply.

Manuscript Instructions

"Extended Data figures are mentioned in the text but were not provided. Please either remove the reference(s) or provide the figure/table.” We double checked and all Extended Data tables with the detailed statistical analysis were provided.

"If your article is accepted for publication, the Extended Data will need to be relabeled as Figure 1-1, Figure 1-2, Table 1-1, etc., so they indicate which figure they are supporting (i.e. Extended Data table supporting Figure 5 labeled as Figure 5-1).” We have double checked and all Extended data are labeled correctly (Figure 1-1, etc...).

Synthesis of Reviews

Computational Neuroscience Model Code Accessibility Comments for Author (Required): N/A

Synthesis Statement for Author (Required):

"This is a strong manuscript and an important advance by establishing relationships between frontal cortex and the hippocampus during olfactory learning with the end goal of understanding if their coupled oscillations may contribute to successful odor learning and discrimination performance. Studies looking into frontal cortex activity in context of olfaction are rare and on that feature alone this manuscript can be seen as an advance. yet here the authors also establish links between the frontal cortex and hippocampus and how their within and between structure dynamics and coupling may lend themselves to successful odor discrimination learning. However, as noted below the authors should elaborate on the KO mice performance and examine the perspective that just because there is a statistically significant difference, this doesn't necessarily mean a functional difference.”

Thank you for the relevant comment that we address below.

Specific comments:

"I found the first half of the paper in WT mice to be strong and of fundamental importance / significance. I do though have a question I'd appreciate the authors to dive into with regards to the latter half of the manuscript which focuses on the possible changes in network dynamics and how this may relate to behavior in the CamKiia KO mice. From my view, the KO and Het mice are very strong performers behaviorally - both in terms of learning the task and also in their discrimination performance. WT and KO mice differed, on average, by <2% in their % of correct responding and no mice performed <85% correct. A <2% reduction in performance in this task would translate into ∼1 additional error out of 10 blocks of performance in a session. And while the statistics do indicate significance, I think stepping back from this result I would interpret these data more-so from the glass is half full lens - that behavioral performance is quite intact and that you do not need CamKiia to engage in quite impressive olfactory discriminations. Yet the data are interpreted (correctly, based on the statistical outcome) that there is a reduction in discrimination performance and then later in the paper this used as a way to correlate the many interesting changes in some of the LFP metrics observed in the KO and Het mice. As the authors discuss, there are many effects of globally reduced CamKiia expression that other groups have published, on both behavior and physiology. So, given that in these mice there are alterations in several mPFC and CA1 LFP metrics, yet despite this behavior is impressively strong relative to what would see in a highly perturbed state, what does this mean about the functional significance of the LFP metrics reported herein?"

This is a relevant comment. We have added a paragraph to the discussion:

Finally, there was a robust decrease of ∼30% in the accuracy for decoding contextual odorant identity from tPRP for the CaMKIIα KO mouse (Figure 11D), but the decrease in behavioral performance in CaMKIIα KO was small (∼1.5%, Figure 9B). The small change in behavioral performance raises the question whether CaMKIIα plays a minor role in learning in the go-no go task. It is possible that CaMKIIα is indeed not involved in olfactory go-no go learning. On the other hand, studies of the role of CaMKIIα in learning and memory indicate that in the CaMKIIα KO there may be compensatory effects from the activation of other isoforms (Zalcman et al., 2018) as well as compensatory developmental changes in molecular mechanisms or circuits involved in learning in the CaMKIIα KO.

Minor:

"Line 317, when first introducing MI in the results, the authors may want to adjust “measure of how localized high frequency firing is within the phase of theta” to instead say “measure of how localized high frequency OSCILLATION is within phase of theta” since of course here the comparison to the carrier signal is an oscillation, not spikes.”

We have made the change.

"Could the authors comment upon how temporally aligned the lick signal is relative to the physiology signals in their set-up? From the methods it seems the licking in this set-up is acquired by a different DAQ from the Intan system used for the LFPs and this may impose some forward or lag (even if just a few ms) into the lick signal relative to the LFPs. Or was the lick contact sensor 0ms lag when relayed into the Intan board? How was possible lag in the matching of these signals accounted for when analyzing licking-triggered/related LFPs?"

The lick signal was recorded by the INTAN at 20 kHz in parallel with the electrode recording. We have added this sentence to the methods: The lick signal was recorded by the INTAN board in parallel with electrode recordings.

"Table 2 would benefit from a footnote to help readers know what the total number of mice from each genotype was which contributed data to the study (of the 18 in total). A quick glance at the table may give the impression otherwise that there were many mice used.”

We added a footnote to what is now Table 3: Note: We used a total of 18 mice: 6 WT, 7 Het and 5 KO, but, as shown in column 2, not all mice were tested with all odorant pairs

"Might the authors be able to comment in the methods regarding approximately what layer(s) the tetrode tips were localized? Deep or superficial IL for instance?"

In the revised manuscript we indicate that the electrodes were targeted to dorsal CA1 and deep IL.

"Just a note that Cansler 2021 is published now, PMID: 35511680”.

Thank you, the reference has been updated.

"There is a “key resources” table in the Materials and Methods. It could be 'new' Table 1, but as is does not have any formal nomenclature linking it in the manuscript.”

We made this Table 1 and we cited it at the beginning of Materials and Methods.
